# Epidemiology of COVID-19 in Tehran, Iran: A Cohort Study of Clinical Profile, Risk Factors, and Outcomes

**DOI:** 10.1155/2022/2350063

**Published:** 2022-05-10

**Authors:** Hamidreza Hatamabadi, Tahereh Sabaghian, Amir Sadeghi, Kamran Heidari, Seyed Amir Ahmad Safavi-Naini, Mehdi Azizmohammad Looha, Nazanin Taraghikhah, Shayesteh Khalili, Keivan Karrabi, Afsaneh Saffarian, Saba Shahsavan, Hossein Majlesi, Amirreza Allahgholipour Komleh, Saba Hatari, Nadia Zameni, Saba Ilkhani, Shideh Moftakhari Hajimirzaei, Aydin Ghaffari, Mohammad Mahdi Fallah, Reyhaneh Kalantar, Nariman Naderi, Parnian Bahmaei, Naghmeh Asadimanesh, Romina Esbati, Omid Yazdani, Fatemeh Shojaeian, Zahra Azizan, Nastaran Ebrahimi, Fateme Jafarzade, Amirali Soheili, Fatemeh Gholampoor, Negarsadat Namazi, Ali Solhpour, Tannaz Jamialahamdi, Mohamad Amin Pourhoseingholi, Amirhossein Sahebkar

**Affiliations:** ^1^Department of Emergency Medicine, School of Medicine, Imam Hossein Hospital, Shahid Beheshti University of Medical Sciences, Tehran, Iran; ^2^Chronic Kidney Disease Research Center (CKDRC), Imam Hossein Hospital, Shahid Beheshti University of Medical Sciences, Tehran, Iran; ^3^Gastroenterology and Liver Diseases Research Center, Research Institute for Gastroenterology and Liver Diseases, Shahid Beheshti University of Medical Sciences, Tehran, Iran; ^4^Skull Base Research Center, Shohada-e-Tajrish Hospital, Shahid Beheshti University of Medical Sciences, Tehran, Iran; ^5^School of Medicine, Shahid Beheshti University of Medical Sciences, Tehran, Iran; ^6^Basic and Molecular Epidemiology of Gastrointestinal Disorders Research Center, Research Institute for Gastroenterology and Liver Diseases, Shahid Beheshti University of Medical Sciences, Tehran, Iran; ^7^Department of Internal Medicine, School of Medicine, Imam Hossein Hospital, Shahid Beheshti University of Medical Sciences, Tehran, Iran; ^8^Department of Emergency Medicine, Shohadaye Tajrish Hospital, Shahid Beheshti University of Medical Sciences, Tehran, Iran; ^9^Department of Internal Medicine, Loghman Hakim Hospital, Shaheed Beheshti University of Medical Sciences, Tehran, Iran; ^10^Student Research Committee, School of Nursing and Midwifery, Shahid Beheshti University of Medical Sciences, Tehran, Iran; ^11^Students Research Committee, School of Medicine, Shahid Beheshti University of Medical Sciences, Tehran, Iran; ^12^University of Florida, Department of Anesthesiology, USA; ^13^Surgical Oncology Research Center, Mashhad University of Medical Sciences, Mashhad, Iran; ^14^Applied Biomedical Research Center, Mashhad University of Medical Sciences, Mashhad, Iran; ^15^Biotechnology Research Center, Pharmaceutical Technology Institute, Mashhad University of Medical Sciences, Mashhad, Iran; ^16^Department of Biotechnology, School of Pharmacy, Mashhad University of Medical Sciences, Mashhad, Iran

## Abstract

**Background:**

The outbreak of coronavirus disease 2019 (COVID-19) dates back to December 2019 in China. Iran has been among the most prone countries to the virus. The aim of this study was to report demographics, clinical data, and their association with death and CFR.

**Methods:**

This observational cohort study was performed from 20th March 2020 to 18th March 2021 in three tertiary educational hospitals in Tehran, Iran. All patients were admitted based on the WHO, CDC, and Iran's National Guidelines. Their information was recorded in their medical files. Multivariable analysis was performed to assess demographics, clinical profile, outcomes of disease, and finding the predictors of death due to COVID-19.

**Results:**

Of all 5318 participants, the median age was 60.0 years, and 57.2% of patients were male. The most significant comorbidities were hypertension and diabetes mellitus. Cough, dyspnea, and fever were the most dominant symptoms. Results showed that ICU admission, elderly age, decreased consciousness, low BMI, HTN, IHD, CVA, dialysis, intubation, Alzheimer disease, blood injection, injection of platelets or FFP, and high number of comorbidities were associated with a higher risk of death related to COVID-19. The trend of CFR was increasing (WPC: 1.86) during weeks 25 to 51.

**Conclusions:**

Accurate detection of predictors of poor outcomes helps healthcare providers in stratifying patients, based on their risk factors and healthcare requirements to improve their survival chance.

## 1. Introduction

Severe acute respiratory syndrome coronavirus 2 (SARS-CoV-2) was officially announced as a pandemic and public health emergence following the first case detected in China in December 2019 and spread rapidly around the world [1]. At the outset, fever and respiratory symptoms were considered as the major symptoms of this novel virus [2]. Over time, the virus caused several clinical manifestations varying from asymptomatic or mild constitutional symptoms to life-threatening conditions leading to hospitalization and even death [3].

Iran has been among the most prone countries to the virus, especially in the Middle East [4–7]. Approximately 3 851 162 COVID-19 patients and 90 344 deaths (mortality rate: 2.34%) have been recorded in Iran until July 30, 2021 [8].

The sudden rise in requisition for healthcare services brings an overload to private and public health systems that require urgent attention to improve optimal services to COVID-19 patients. As a result, the evaluation of the most common risk factors of mortality, length of hospital stay, and outcome of COVID-19 has become crucial to guide healthcare professionals in decision-making and get the most out of their skills and facilities to immediately detect cases and evaluate the course of infection and to improve treatment outcomes and reduce virus transmission and mortality rates [9–14]. Multiple studies have reported the association of patients' medical records such as demographics, clinical manifestations, and disease outcome, to the COVID-19 pandemic progression to recognize the risk factors of hospitalization and mortality due to SARS-CoV-2 [15–19]. A review article of Wynants et al. demonstrated the relation of age, sex, comorbidities, and serum biomarkers, such as C-reactive protein (CRP), creatinine, lymphocyte count, and lactate dehydrogenase (LDH) with increased mortality risk [18].

Obviously, the patients' epidemiology varies in different countries in the matters of population demographic data, genetic, the prevalence of comorbidities, and health care systems [20]. To the best of our knowledge, limited studies estimated the case fatality rate (CFR) of this outbreak in Iran. The case fatality rate is a value of the ability of a virus to damage a host and represents the proportion of death from a specified disease among all diagnosed cases during the exact period of time [21]. The CFR is one of the substantial parameters to estimate the basic epidemiological features of the outbreak and the severity of disease and is also essential for public health services in approaches to reduce the risk of disease [22]. Our study evaluates the CFR of COVID-19 since the outset of the pandemic in Iran.

The purpose of this retrospective study was to investigate the epidemiology, clinical outcomes, therapeutic protocols, and the potential risk factors of in-hospital mortality of the COVID-19 cases from academic and referral health care centers in Tehran, the most populous city in Iran, since the outbreak of COVID-19 pandemic. Besides, this study is aimed at calculating CFR to hopefully provide successful guidelines to block transmission of SARS-CoV-2, early detection of severe cases, and perform effective therapeutic guidelines.

## 2. Patients and Methods

### 2.1. Study Design and Data Collection

In this retrospective study, confirmed COVID-19 patients admitted to three university hospitals (including Taleghani hospital, Imam Hussein hospital, and Shohadaye Tajrish hospital) in Tehran, Iran, were enrolled from 20 March 2020 until 18 March 2021. Real-time polymerase chain reaction (RT-PCR) of nasopharyngeal or oropharyngeal swab samples was performed to confirm COVID-19 cases on the first days of admission. The medical team gathered demographics, comorbidities, triage vital signs, patient outcomes, inpatient treatment protocol, and laboratory data through the hospital information system.

### 2.2. Patient's Characteristic, Treatment, and Outcome

A medical team collected demographic data (age, sex, body mass index), presenting symptoms, symptom onset to admission interval (days), comorbidities, habitual history (smoking, alcohol, opium, hookah), and triage vital signs (pulse rate, respiratory rate, blood pressure, oxygen saturation without supplementary oxygen, oxygen saturation with supplementary oxygen, body temperature measure by infrared thermometer) from electronic medical records. Inpatient medication and treatment protocol were retrieved from the nursing notes. Outcomes were determined as death versus survived, ICU admission versus ward admission, invasive mechanical ventilation, and length of admission.

### 2.3. Laboratory Data

Laboratory values during the admission were gathered from the hospital information system and sorted using the Python program (Python Software Foundation. Python Language Reference, version 2.7. Available at http://www.python.org). Some parameters were gathered during the first six days of admission, if available. For other laboratory data, the earliest valid value is considered.

### 2.4. Statistical Analysis

Descriptive statistics were presented using mean ± SD and frequency (percentage) for continuous and categorical data, respectively. Bar charts were also used to display summary statistics such as frequency or percentage by demographic or outcome variables. In order to examine the relationship between outcome and explanatory variables, Pearson chi-square and Fisher exact tests were used. The measure of association between outcome and variables was assessed by Cramer's V and Eta. The Kaplan-Meier estimator was used to estimate the survival function. The logrank test was used to compare the risk of death in different categories of a variable. Weekly percent change (WPC) has been used to evaluate the rate of change or trend in CFR each week between the 3rd week and the 50th week of the study. All analyzes were performed by SPSS (version 26), R (4.0.2), and Joinpoint regression (4.9.0.0). *p* values less than 0.05 were regarded as statistically significant.

### 2.5. Ethics Statement

The study was approved by the Institutional Review Board (IRB) of the Shahid Beheshti University of Medical Sciences (IR.SBMU.RIGLD.REC.004), and IRB exempted this study from informed consent. Data were anonymized before analysis; patients' confidentiality and data security were concerned at all levels, and the study was completed under the Helsinki Declaration (2013) guidelines.

## 3. Results

### 3.1. Demographic, Clinical Characteristics, and Outcome of Patients

**Figure 1:**
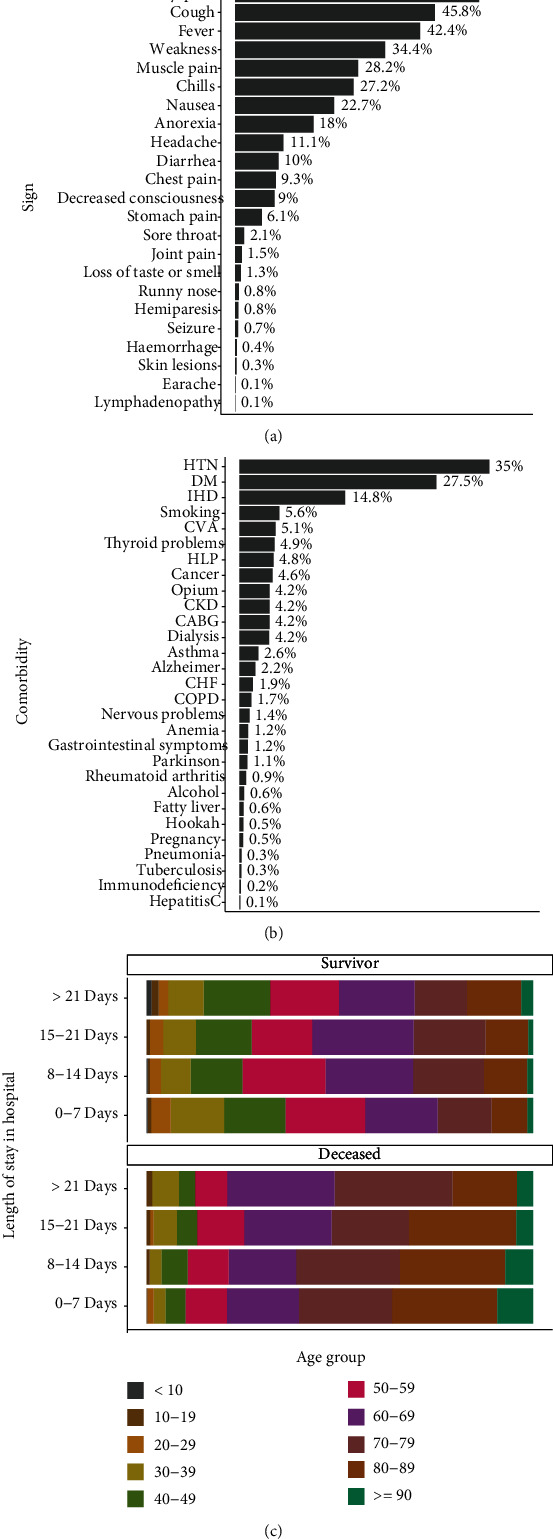
The percentage of (a) sign, (b) comorbidity, and (c) deceased patients by age group and length of stay in hospital.

A total of 5 318 patients were included in this study (3 042 males and 2 276 females) with a median age of 60.0 (Q1, Q3, 46.0, 74.0) years old. Patients' clinical characteristics and outcomes were summarized in Table 1. Twenty-one percent (*n* = 1112) of patients with COVID-19 were deceased. The median age among deceased patients was significantly higher than that of in the survivor group (73.0 vs. 57.0 years, *p* < 0.001). The association between sex and death was not significant (*p* = 0.151). Among variables with significant relation with death, the strength of the relationship between death and variables including intubation (Cramer's V = 0.45), oxygen saturation (Eta = 0.32), O2 saturation with ventilator (Eta = 0.30), age (Cramer's V = 0.30), and decreased consciousness (Cramer's V = 0.27) was highest. As shown in Table 1 and Figures 1(a) and 1(b), the main symptoms at admission were dyspnea, cough, fever, weakness, muscle pain, chills, and nausea, respectively. HTN, DM, and IHD were common comorbidities. The age percentage by death status and length of stay in hospital is shown Figure 1(c). Accordingly, among the patients who died, those older than 60 years accounted for approximately 75% of the cases in various categories of the length of hospital stay.

### 3.2. Clinical Laboratory Data

In the next step, we investigated the ranges of laboratory data between deceased and survived patients, which are summarized in Table 2 (see Table S1 in the Supplementary File).

### 3.3. Drug Being Tested to Treat COVID-19 for Hospitalized Patients

The drugs used to treat patients with COVID-19 in hospitals are presented in Table 3 and Figure S1-A in the Supplementary File. Overall, 835 patients had received the remdesivir, and the death rate was the 29.0%. In addition, the death rate of Dexamethasone and Clexane was 23.0% and 17.4%, respectively. As shown in Figure S1-B in the Supplementary File, almost all drugs were used less in the last 3 months of the study than in the third trimester.

### 3.4. Survival Rate of COVID-19 Patients

**Figure 2:**
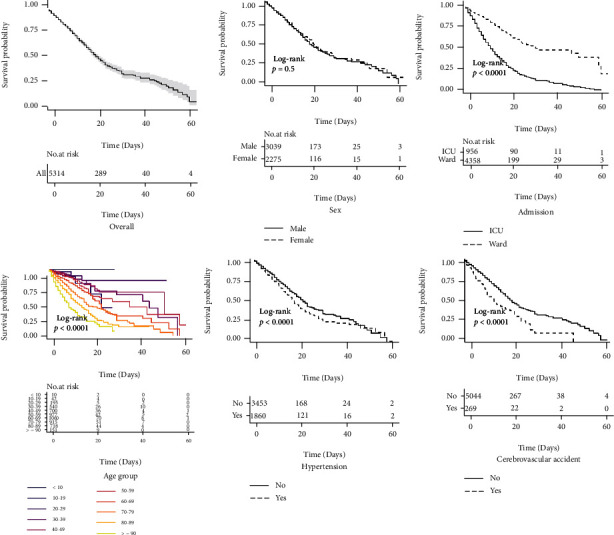
The Kaplan-Meier survival time by demographic variables.

The survival rate of COVID-19 patients and its risk factors were assessed using Kaplan-Meier estimator (Figure 2 and Figure S2 in the Supplementary File). Accordingly, the survival rates of patients in the first, second, and third weeks of hospitalization were about 0.85, 0.65, and 0.50, respectively. The risk of death was not different between men and women (*p* = 0.500), but it was significantly associated with several factors as shown in Figure 2, including ICU admission, older age, HTN, and CVA.

### 3.5. The CFR of COVID-19 Patients

**Figure 3:**
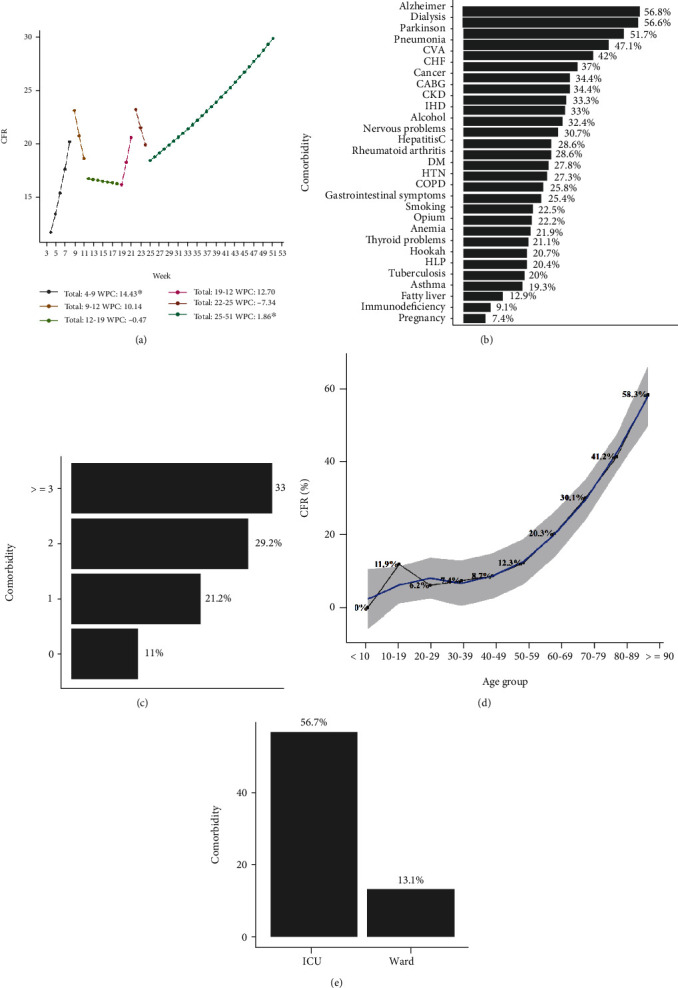
The case fatality rate of COVID-19 patients.

As shown in Figure 3(a), the CFR of COVID-19 has changed over time. Overall, five joinpoints found in weeks of 9, 12, 19, 22, and 25. In addition, the last trend of CFR was upward and significant (WPC: 14.43% for weeks of 4-9; WPC: 1.86% for weeks of 25-51). According to Figure 3(b), CFR among COVID-19 patients with comorbidities of Alzheimer, dialysis, Parkinson, pneumonia, and CVA were higher than 40%. Based on Figure 3(c), the higher number of comorbidities was associated with higher CFR. As shown in Figure 3(d), the CFR has grown linearly with a slope of 10% from patients aged 50 years and older.  Figure 3(e) shows that the CFR for patients admitted to the ICU was 3.1 times higher than that in the general ward.

## 4. Discussion

According to our data, 5 318 COVID-19 patients were admitted to three tertiary university hospitals in Tehran, Iran, from 20 March 2020 to 18 March 2021. To the best of our knowledge, this is the largest national sample of COVID-19 inpatients with detailed information in one of the remarkable centers of SARS-CoV-2 in Iran. Our findings include detailed demographics, clinical characteristics, paraclinical data, therapeutic agents, and their association with survival rate and CFR.

The majority of cases were men with the median age of 60 years suffering from hypertension and diabetes, which was in line with China, USA, and Italy patterns [23, 24]. The most predominant symptoms were dyspnea (55.9%), cough (45.8%), fever (42.4%), and weakness (34.4%) which were consistent with Rivera-Izquierdo et al. [25] and Guan et al. [26]. 21% of patients were deceased in hospital, which was similar to Germany and France [20], but lower than UK with 39% of mortality [27]. Definitely, this rate could vary, regarding to significant differences between countries in epidemiology, health care systems, and lengths of follow-up. The significant risk factors of death related to COVID-19 were aging, loss of consciousness, the need for intubation and low O2 saturation, and high ranges of WBC, BUN, LDH, IL-6, pro-BNP, and HCO3, which are consistent with prior reports [28–30]. In accordance with Rosenthal et al. study, patients older than 65 years accounted for more than 75% of all in-hospital mortality [31]. Similarly, Cummings et al. reported older age, cardiopulmonary disease, and higher ranges of CRP, and liver and renal tests as predictors of poor progression [32]. High levels of serum creatinine and urea could be due to direct kidney damage or fluid imbalance, and also leukocytosis might be a sign of bacterial superinfection. Similar to China [33] and Italy [34], hypertension and diabetes were associated with poor prognosis. The same as our study, Aggarwal et al. reported that the severity of COVID-19 among patients with cerebrovascular disease is higher [35]. Deceased cases had higher range of blood pressure, pulse rate, respiratory rate, and lower oxygen saturation compared to survivors. The data showed that abnormal vital signs could be predictors of severity. In contrary to Brazilian study [36], we had a weak relationship between age and length of hospital stay since elderly tend to stay more time in the hospital, and on the other hand, younger patients had a higher chance to recover from COVID-19 than older cases.

Remdesivir was administered to 15.72% of cases and had a significant role in their survival. The US Food and Drug Administration approved an emergency use of remdesivir for critical cases of COVID-19 on May 1, 2020 [37, 38]. Enoxaparin and heparin were used in nearly 85% of cases and had a beneficial effect due to prophylaxis and treatment of thrombosis and thrombophilia triggered by COVID-19 [39]. Another challenging drug is Dexamethasone with presented positive results similar to several studies by suppressing the proinflammatory storm of cytokines and chemokines [40]. Guidelines of the UK chief medical officers, the European Medicines Agency, the World Health Organization, and the National Institutes of Health in the United States have approved the use of glucocorticoids in hospitalized cases requiring oxygen support [41–43]. In order to evaluate the impact of each therapeutic agent, more researches are required, whereas these effects are evaluated beside several factors in this study.

The most important features of this study were the estimation of survival rate, CFR of COVID-19 inpatients, and their association with epidemiological factors. Our findings confirm that survival rate of COVID-19 inpatients is exclusively low for older cases requiring ICU admission and intubation and with underlying comorbidities including HTN, IHD, and CVA. These data was in line with a study from Italy and England [44, 45]. The trend of CFR was increasing (WPC: 1.86) during weeks 25 to 51, which is similar to Yemen [46]. This pattern might be due to more accurate recording of cases medical data or the hypothesis that gradually SARS-CoV-2 turns into more invasive variants. In contrary to our study, the rCFR is declining gradually over time in England and New York, which could be attributed to increased detection of asymptomatic or mild cases, improvements in medical management of severely ill patients, and increased public awareness [45, 47]. The CFR varies among different countries, since the calculations, PCR testing, and healthcare services are different. There was significant relation among CFR with aging and comorbidities, especially DM, dialysis, and cancer. Actually, older people had more comorbidities and compromised immune systems and are more vulnerable to infectious disease [48]. Also, these results could be a clue that exacerbation of preexisting conditions due to SARS-CoV-2 increases the death rate of COVID-19 in cases with comorbidities [49]. Perone reported the association of environmental, demographics, and healthcare factors with CFR [50]. Comprehensive estimation of CFR could be served as a theory for successful control of COVID-19 in Iran, by studying the future patterns of CFR.

This study had some strength points. First, the important variables related to the mortality of COVID-19 patients were determined using effect size indices, and the survival rate of patients in different categories of these variables was assessed. Second, the most common symptoms, comorbidities, and prescribed medications were identified among patients with COVID-19, and CFR was reported in patients with various comorbidities and medications. The trends of CFR were evaluated during the study period by age and sex. Fourth, all laboratory data of COVID-19 patients were included in this study. However, the study had some limitations. First, all of our cases were hospitalized, which is a bias to outpatients, so these results could be overestimated and needs further studies to provide a standard approach for accurate and acceptable guidelines. Second, follow-up after discharge was not performed in this study, so we could not be able to include postdischarge deceased cases. Third, there was no data about noninvasive respiratory support including CPAP and NIV.

## 5. Conclusions

Since SARS-CoV-2 is a novel virus and the pandemic is still alive, we provide a large cohort study to evaluate demographics and clinical profile and their association with mortality. Older patients and cases with comorbidities are at a higher risk for developing complications from COVID-19 infection and even death. Considering the increasing trend of CFR, it is crucial to guide healthcare providers in decision-making and get the most out of their skills and facilities to immediately detect at-risk cases and evaluate the course of infection, to improve therapeutic protocols and reduce virus transmission and mortality rates.

## Figures and Tables

**Table 1:** Clinical characteristics and outcomes of patients hospitalized for treatment of COVID-19 in hospitals in Tehran.

Variables		Total (*n* = 5318)	Survivor (*n* = 4204)	Deceased (*n* = 1112)	Cramer's V/Eta	*p* value
Age		60.0 (46.0, 74.0)	57.0 (43.0, 70.0)	73.0 (61.0, 83.0)	0.30	<0.001
BMI		26.3 (23.9, 29.4)	26.4 (24.0, 29.6)	26.0 (22.9, 29.4)	0.05	0.028
Sex	Male	3042 (57.20)	2383 (56.68)	657 (59.08)	0.02	0.151
	Female	2276 (42.80)	1821 (43.32)	455 (40.92)		
Cough	No	2884 (54.23)	2227 (52.97)	656 (58.99)	0.05	<0.001
	Yes	2434 (45.77)	1977 (47.03)	456 (41.01)		
Dyspnea	No	2342 (44.04)	1906 (45.34)	436 (39.21)	0.05	<0.001
	Yes	2975 (55.94)	2297 (54.64)	676 (60.79)		
Fever	No	3064 (57.62)	2378 (56.57)	685 (61.60)	0.04	0.003
	Yes	2254 (42.38)	1826 (43.43)	427 (38.40)		
Chills	No	3872 (72.81)	3023 (71.91)	848 (76.26)	0.014	0.004
	Yes	1445 (27.17)	1180 (28.07)	264 (23.74)		
Muscle pain	No	3818 (71.79)	2921 (69.48)	895 (80.49)	0.1	<0.001
	Yes	1498 (28.17)	1282 (30.49)	216 (19.42)		
Weakness	No	3486 (65.55)	2821 (67.10)	664 (59.71)	0.06	<0.001
	Yes	1829 (34.39)	1381 (32.85)	447 (40.20)		
Decreased consciousness	No	4836 (90.94)	3990 (94.91)	844 (75.90)	0.27	<0.001
	Yes	481 (9.04)	213 (5.07)	268 (24.10)		
Sore throat	No	5207 (97.91)	4110 (97.76)	1095 (98.47)	0.02	0.142
	Yes	111 (2.09)	94 (2.24)	17 (1.53)		
Runny nose	No	5273 (99.15)	4169 (99.17)	1102 (99.10)	0	0.829
	Yes	45 (0.85)	35 (0.83)	10 (0.90)		
Loss of taste or smell	No	5247 (98.66)	4138 (98.43)	1107 (99.55)	0.04	0.004
	Yes	71 (1.34)	66 (1.57)	5 (0.45)		
Nausea	No	4109 (77.27)	3202 (76.17)	905 (81.38)	0.05	<0.001
	Yes	1208 (22.72)	1001 (23.81)	207 (18.62)		
Anorexia	No	4358 (81.95)	3437 (81.76)	921 (82.82)	0.01	0.427
	Yes	958 (18.01)	765 (18.20)	191 (17.18)		
Diarrhea	No	4788 (90.03)	3755 (89.32)	1031 (92.72)	0.05	0.001
	Yes	530 (9.97)	449 (10.68)	81 (7.28)		
Chest pain	No	4821 (90.65)	3784 (90.01)	1035 (93.08)	0.04	0.002
	Yes	497 (9.35)	420 (9.99)	77 (6.92)		
Lymphadenopathy	No	5315 (99.94)	4201 (99.93)	1112 (100.00)	0.01	0.373
	Yes	3 (0.06)	3 (0.07)	0 (0.00)		
Skin lesions	No	5300 (99.66)	4196 (99.81)	1102 (99.10)	0.05	<0.001
	Yes	18 (0.34)	8 (0.19)	10 (0.90)		
Joint pain	No	5237 (98.48)	4140 (98.48)	1095 (98.47)	0	0.988
	Yes	81 (1.52)	64 (1.52)	17 (1.53)		
Headache	No	4729 (88.92)	3686 (87.68)	1041 (93.62)	0.08	<0.001
	Yes	588 (11.06)	517 (12.30)	71 (6.38)		
Stomach pain	No	4993 (93.89)	3946 (93.86)	1045 (93.97)	0	0.89
	Yes	325 (6.11)	258 (6.14)	67 (6.03)		
Earache	No	5311 (99.87)	4198 (99.86)	1111 (99.91)	0.01	0.666
	Yes	7 (0.13)	6 (0.14)	1 (0.09)		
Haemorrhage	No	5298 (99.62)	4193 (99.74)	1103 (99.19)	0.04	0.008
	Yes	20 (0.38)	11 (0.26)	9 (0.81)		
Hemiparesis	No	3976 (74.76)	3128 (74.41)	847 (76.17)	0.01	0.391
	Yes	41 (0.77)	30 (0.71)	11 (0.99)		
Pregnancy	No	3991 (75.05)	3134 (74.55)	856 (76.98)	0.03	0.076
	Yes	27 (0.51)	25 (0.59)	2 (0.18)		
Smoking	No	5020 (94.40)	3973 (94.51)	1045 (93.97)	0.01	0.494
	Yes	298 (5.60)	231 (5.49)	67 (6.03)		
Alcohol	No	5284 (99.36)	4181 (99.45)	1101 (99.01)	0.02	0.100
	Yes	34 (0.64)	23 (0.55)	11 (0.99)		
Opium	No	5092 (95.75)	4029 (95.84)	1061 (95.41)	0.01	0.619
	Yes	225 (4.23)	175 (4.16)	50 (4.50)		
Hookah	No	5289 (99.45)	4181 (99.45)	1106 (99.46)	0	0.976
	Yes	29 (0.55)	23 (0.55)	6 (0.54)		
HTN	No	3456 (64.99)	2850 (67.79)	604 (54.32)	0.12	<0.001
	Yes	1861 (34.99)	1353 (32.18)	508 (45.68)		
IHD	No	4532 (85.22)	3677 (87.46)	853 (76.71)	0.12	<0.001
	Yes	786 (14.78)	527 (12.54)	259 (23.29)		
CABG	No	5094 (95.79)	4057 (96.50)	1035 (93.08)	0.07	<0.001
	Yes	224 (4.21)	147 (3.50)	77 (6.92)		
CHF	No	5218 (98.12)	4141 (98.50)	1075 (96.67)	0.06	<0.001
	Yes	100 (1.88)	63 (1.50)	37 (3.33)		
Asthma	No	5178 (97.37)	4091 (97.31)	1085 (97.57)	0.01	0.63
	Yes	140 (2.63)	113 (2.69)	27 (2.43)		
COPD	No	5228 (98.31)	4138 (98.43)	1088 (97.84)	0.02	0.248
	Yes	89 (1.67)	66 (1.57)	23 (2.07)		
DM	No	3852 (72.43)	3145 (74.81)	705 (63.40)	0.1	<0.001
	Yes	1465 (27.55)	1058 (25.17)	407 (36.60)		
Pneumonia	No	5301 (99.68)	4195 (99.79)	1104 (99.28)	0.04	0.008
	Yes	17 (0.32)	9 (0.21)	8 (0.72)		
CVA	No	5048 (94.92)	4047 (96.27)	999 (89.84)	0.12	<0.001
	Yes	269 (5.06)	156 (3.71)	113 (10.16)		
Gastrointestinal symptoms	No	5255 (98.82)	4157 (98.88)	1096 (98.56)	0.01	0.379
	Yes	63 (1.18)	47 (1.12)	16 (1.44)		
CKD	No	5093 (95.77)	4054 (96.43)	1037 (93.26)	0.06	<0.001
	Yes	225 (4.23)	150 (3.57)	75 (6.74)		
Rheumatoid arthritis	No	5269 (99.08)	4169 (99.17)	1098 (98.74)	0.02	0.186
	Yes	49 (0.92)	35 (0.83)	14 (1.26)		
Cancer	No	5047 (94.90)	4028 (95.81)	1017 (91.46)	0.07	<0.001
	Yes	247 (4.64)	162 (3.85)	85 (7.64)		
HLP	No	5062 (95.19)	4000 (95.15)	1060 (95.32)	0	0.831
	Yes	255 (4.80)	203 (4.83)	52 (4.68)		
Hepatitis C	No	5310 (99.85)	4198 (99.86)	1110 (99.82)	0.01	0.619
	Yes	7 (0.13)	5 (0.12)	2 (0.18)		
Thyroid problems	No	5048 (94.92)	3991 (94.93)	1055 (94.87)	0	0.949
	Yes	261 (4.91)	206 (4.90)	55 (4.95)		
Immunodeficiency	No	5307 (99.79)	4194 (99.76)	1111 (99.91)	0.01	0.334
	Yes	11 (0.21)	10 (0.24)	1 (0.09)		
Seizure	No	5255 (98.82)	4156 (98.86)	1097 (98.65)	0.01	0.570
	Yes	63 (1.18)	48 (1.14)	15 (1.35)		
Tuberculosis	No	5303 (99.72)	4192 (99.71)	1109 (99.73)	0	0.930
	Yes	15 (0.28)	12 (0.29)	3 (0.27)		
Anemia	No	5252 (98.76)	4153 (98.79)	1097 (98.65)	0	0.484
	Yes	64 (1.20)	50 (1.19)	14 (1.26)		
Fatty liver	No	5287 (99.42)	4177 (99.36)	1108 (99.64)	0.02	0.271
	Yes	31 (0.58)	27 (0.64)	4 (0.36)		
Nervous problems	No	5235 (98.44)	4146 (98.62)	1087 (97.75)	0.03	0.036
	Yes	75 (1.41)	52 (1.24)	23 (2.07)		
Parkinson	No	5260 (98.91)	4176 (99.33)	1082 (97.30)	0.08	<0.001
	Yes	58 (1.09)	28 (0.67)	30 (2.70)		
Alzheimer	No	5200 (97.78)	4153 (98.79)	1045 (93.97)	0.13	<0.001
	Yes	118 (2.22)	51 (1.21)	67 (6.03)		
Dialysis	No	5097 (95.84)	4108 (97.72)	987 (88.76)	0.18	<0.001
	Yes	221 (4.16)	96 (2.28)	125 (11.24)		
Blood injection	No	4791 (90.09)	3909 (92.98)	881 (79.23)	0.19	<0.001
	Yes	522 (9.82)	292 (6.95)	229 (20.59)		
Injection of platelets or fresh frozen plasma (FFP)	No	5188 (97.56)	4145 (98.60)	1041 (93.62)	0.13	<0.001
	Yes	130 (2.44)	59 (1.40)	71 (6.38)		
Intubation	No	4883 (91.82)	4126 (98.14)	755 (67.90)	0.45	<0.001
	Yes	432 (8.12)	75 (1.78)	357 (32.10)		
Number of days hospitalized in the hospital emergency department	—	1.0 (1.0, 1.0)	1.0 (1.0, 1.0)	1.0 (1.0, 1.0)	0.04	0.159
Number of days hospitalized in the hospital general department	—	5.0 (2.0, 9.0)	5.0 (2.0, 9.0)	4.0 (1.0, 8.0)	0.03	<0.001
Number of days hospitalized in the hospital ICU department	—	4.0 (2.0, 8.0)	5.0 (2.0, 9.0)	4.0 (1.0, 8.0)	0.02	0.035
Oxygen saturation	—	90.0 (85.0, 93.0)	90.0 (86.0, 94.0)	85.0 (76.0, 90.0)	0.32	<0.001
O2 saturation with ventilator	—	95.0 (92.0, 98.0)	96.0 (93.0, 98.0)	93.0 (88.0, 97.0)	0.30	<0.001
Pulse rate	—	85.0 (80.0, 95.0)	85.0 (80.0, 93.0)	88.0 (80.0, 100.0)	0.08	<0.001
Diastolic pressure	—	80.0 (70.0, 80.0)	80.0 (70.0, 80.0)	75.0 (70.0, 80.0)	0.02	0.007
Systolic pressure	—	120.0 (110.0, 130.0)	120.0 (110.0, 130.0)	120.0 (100.0, 130.0)	0.03	0.001
Respiratory rate	—	18.0 (17.0, 20.0)	18.0 (17.0, 20.0)	19.0 (18.0, 22.0)	0.10	<0.001
Body temperature	—	37.0 (36.9, 37.5)	37.0 (36.9, 37.5)	37.0 (36.8, 37.5)	0.01	0.653

The Cramer's V test was used to measure the association between categorical variables and status. The value of Cramer's V indicates how strongly two categorical variables are associated, giving a value between 0 and +1. For numeric variables, the Mann–Whitney test was used to compare median values between survivors and deceased cases. Eta was used to measure the association of numeric variables with status, giving a value between 0 and 1. In both Cramer's V and Eta, values close to 1 indicating a high degree of association. The missing values were ignored in calculation of percentages. The median (Q1, Q3) and frequency (%) were used for describing the numeric and categorical variables, respectively.

**Table 2:** Laboratory statistics of COVID-19 patients in Tehran.

Variables		Total (*n* = 5318)	Survivor (*n* = 4204)	Deceased (*n* = 1112)	Cramer's V/Eta	*p* value
WBC (×103/*μ*L)	—	7.3 (5.2, 10.5)	6.9 (5.0, 9.7)	9.1 (6.2, 13.2)	0.17	<0.001
Lymphs (%)	—	15.6 (10.0, 24.9)	17.9 (11.0, 25.4)	10.1 (7.1, 17.1)	0.22	<0.001
NEUT (%)	—	79.5 (70.0, 85.0)	76.9 (68.0, 85.0)	85.0 (77.4, 90.0)	0.23	<0.001
PLT (×103/*μ*L)	—	194.0 (150.0, 255.0)	196.0 (152.0, 254.0)	186.0 (138.5, 259.0)	0.04	<0.001
HB (g/dL)	—	12.4 (10.9, 13.7)	12.5 (11.1, 13.8)	11.9 (10.1, 13.3)	0.12	<0.001
MCV (*μ*m^3^)	—	84.6 (80.5, 88.3)	84.3 (80.4, 88.0)	85.7 (80.7, 89.7)	—	<0.001
BUN (mg/dL)	—	19.0 (13.0, 31.0)	17.0 (12.0, 26.0)	29.0 (18.3, 48.8)	0.29	<0.001
CR (mg/dL)	—	1.1 (1.0, 1.5)	1.1 (0.9, 1.4)	1.4 (1.1, 2.2)	0.19	<0.001
NA (mEq/L)	—	138.0 (135.0, 141.0)	138.0 (135.0, 140.0)	138.0 (135.0, 141.0)	0.04	0.031
K (mEq/L)	—	4.1 (3.8, 4.4)	4.1 (3.8, 4.4)	4.2 (3.9, 4.7)	0.13	<0.001
CA (mg/dL)	—	8.6 (8.1, 9.3)	8.7 (8.2, 9.3)	8.5 (8.0, 9.1)	0.09	<0.001
MG (mEq/L)	—	1.9 (1.7, 2.2)	1.9 (1.7, 2.1)	2.0 (1.8, 2.2)	0.08	<0.001
P (mg/dL)	—	3.5 (2.9, 4.1)	3.4 (2.9, 4.0)	3.8 (3.1, 4.7)	0.22	<0.001
AST (U/L)	—	36.0 (24.0, 55.0)	34.0 (23.4, 50.0)	44.9 (29.0, 72.0)	0.09	<0.001
ALT (U/L)	—	28.0 (18.0, 46.0)	27.1 (18.0, 45.0)	30.0 (18.0, 50.4)	0.07	0.021
ALKP (U/L)	—	185.0 (138.0, 257.0)	181.0 (136.0, 248.0)	205.0 (148.0, 287.0)	0.12	<0.001
BILLT (mg/dL)	—	0.8 (0.6, 1.1)	0.8 (0.6, 1.1)	0.9 (0.6, 1.2)	0.11	<0.001
BILLD (mg/dL)	—	0.3 (0.2, 0.4)	0.3 (0.2, 0.4)	0.4 (0.2, 0.5)	0.13	<0.001
Amylase (U/L)	—	53.0 (38.8, 76.8)	54.0 (40.0, 75.8)	49.9 (34.0, 80.0)	0.0	0.164
LIPASE (U/L)	—	26.0 (19.0, 38.0)	26.0 (19.0, 38.0)	25.0 (17.6, 38.0)	0.01	0.559
TG (mg/dL)	—	120.0 (90.0, 168.0)	119.0 (90.0, 168.0)	123.0 (87.8, 173.0)	0.01	0.957
Cholesterol (mg/dL)	—	130.0 (106.0, 158.0)	133.5 (110.0, 161.0)	119.5 (96.8, 148.0)	0.14	<0.001
HDL (mg/dL)	—	31.0 (28.0, 40.0)	32.0 (28.0, 40.0)	30.1 (26.0, 38.0)	0.04	0.053
LDL (mg/dL)	—	73.0 (54.0, 95.0)	75.0 (58.0, 98.0)	65.0 (48.0, 84.0)	0.14	<0.001
FBS (mg/dL)	—	135.0 (104.0, 194.0)	131.0 (103.0, 188.0)	146.0 (109.8, 207.3)	0.06	0.001
HBA1C (% of total Hb)	—	7.5 (6.4, 9.9)	7.5 (6.4, 10.0)	7.6 (6.4, 9.5)	0.03	0.527
Albumin (g/dL)	—	3.8 (3.4, 4.2)	3.9 (3.5, 4.3)	3.5 (3.1, 3.9)	0.28	<0.001
LDH (U/L)	—	576.0 (439.0, 800.0)	547.5 (421.8, 745.0)	711.0 (520.5, 1072.0)	0.24	<0.001
CRP (mg/L)	—	29.7 (10.5, 69.1)	26.8 (10.0, 64.0)	43.4 (15.0, 86.0)	—	<0.001
ESR (mm/h)	—	34.0 (18.0, 56.0)	32.0 (18.0, 56.0)	36.0 (20.0, 59.0)	0.06	<0.001
Lactate	—	20.0 (15.0, 27.0)	19.1 (15.0, 25.9)	22.0 (16.0, 33.0)	0.20	<0.001
IL6 (pg/mL)	—	25.6 (10.9, 70.2)	18.5 (8.1, 44.8)	46.6 (16.1, 146.0)	0.33	0.004
CPK (U/L)	—	117.0 (63.0, 257.0)	108.0 (61.0, 232.0)	150.0 (77.5, 356.5)	0.08	<0.001
CKMB (U/L)	—	21.0 (14.0, 33.0)	20.0 (14.0, 30.0)	25.0 (17.0, 45.0)	0.12	<0.001
PROBNP (pg/mL)	—	868.0 (173.8, 3792.8)	469.0 (132.0, 2313.0)	3200.0 (894.0, 9987.0)	0.32	<0.001
Procalcitonin (pg/mL)	—	0.4 (0.2, 1.3)	0.3 (0.2, 0.9)	0.9 (0.3, 2.6)	0.08	<0.001
PTT (s)	—	30.0 (25.6, 35.0)	30.0 (25.3, 35.0)	32.0 (26.7, 38.0)	0.09	<0.001
PT (s)	—	13.0 (11.9, 13.7)	13.0 (11.7, 13.3)	13.0 (12.4, 14.6)	0.14	<0.001
INR	—	1.1 (1.0, 1.2)	1.1 (1.0, 1.2)	1.1 (1.0, 1.3)	0.16	<0.001
pH	—	7.4 (7.3, 7.4)	7.4 (7.3, 7.4)	7.4 (7.3, 7.4)	0.08	<0.001
PCO2 (mm Hg)	—	44.3 (38.7, 50.0)	44.6 (39.3, 50.1)	42.7 (36.3, 49.8)	0.04	<0.001
HCO3 (mEq/L)	—	25.8 (22.7, 28.6)	26.2 (23.5, 28.9)	23.8 (20.2, 27.3)	0.20	<0.001
BE (mmol/L)	—	1.6 (-1.6, 4.4)	2.0 (-0.7, 4.6)	-0.4 (-5.2, 3.0)	0.21	<0.001
ANCA (AU/mL)	—	1.5 (0.9, 8.8)	1.6 (1.0, 12.4)	1.0 (1.0, 1.0)	0.27	0.480
CANCA (AU/mL)	—	2.4 (1.8, 4.0)	2.1 (1.4, 3.0)	3.6 (2.7, 6.3)	0.19	0.015
PANCA (AU/mL)	—	2.9 (1.7, 4.5)	2.9 (1.7, 4.4)	2.8 (1.7, 4.8)	0.09	0.883
FDP (mug/mL)	—	6.5 (4.0, 12.0)	5.9 (4.0, 9.4)	12.0 (6.2, 18.0)	0.30	<0.001
Fe (*μ*g/dL)	—	43.0 (25.0, 80.0)	44.0 (25.0, 79.8)	38.5 (24.0, 82.5)	0.00	0.509
Ferritin (ng/mL)	—	361.0 (194.0, 639.9)	340.3 (182.6, 598.6)	456.3 (257.0, 762.0)	—	<0.001
TIBC (*μ*g/dL)	—	260.0 (193.3, 328.3)	269.0 (202.0, 330.0)	236.0 (167.0, 309.5)	0.10	0.002
Total protein (g/dL)	—	5.8 (5.2, 6.5)	6.1 (5.4, 6.7)	5.6 (5.0, 6.2)	0.18	0.007
TSH (*μ*IU/mL)	—	1.0 (0.4, 2.0)	1.1 (0.5, 2.0)	1.0 (0.4, 1.9)	0.01	0.282
T4 (*μ*g/dL)	—	8.1 (6.4, 9.6)	8.4 (6.8, 9.8)	7.1 (5.3, 8.5)	0.24	<0.001
T3(ng/dL)	—	0.9 (0.7, 1.1)	0.9 (0.7, 1.1)	0.8 (0.6, 1.0)	0.17	<0.001
VitD3 (ng/mL)	—	25.1 (15.6, 39.0)	24.5 (15.5, 38.4)	27.6 (17.1, 42.2)	0.04	0.027
IgM (g/L)	—	65.5 (38.5, 112.5)	98.0 (37.8, 127.3)	59.0 (36.5, 65.3)	0.34	0.052
IgG (g/L)	—	1060.5 (835.0, 1394.5)	1073.0 (877.8, 1422.0)	976.5 (700.5, 1256.0)	0.16	0.228
UREA (mg/dL)	—	37.4 (26.9, 56.0)	34.4 (25.0, 48.0)	57.3 (37.3, 88.8)	0.33	<0.001

The Cramer's V test was used to measure the association between categorical variables and status. The value of Cramer's V indicates how strongly two categorical variables are associated, giving a value between 0 and +1. For numeric variables, the Mann–Whitney test was used to compare median values between survivors and deceased cases. Eta was used to measure the association of numeric variables with status, giving a value between 0 and 1. In both Cramer's V and Eta, values close to 1 indicating a high degree of association. The missing values were ignored in calculation of percentages. The median (Q1, Q3) and frequency (%) were used for describing the numeric and categorical variables, respectively. The baseline values of WBC, lymph, NEUT, PLT, HB, MCV, BUN, CR, AST, ALT, LDH, CRP, and UREA were summarized.

**Table 3:** Descriptive statistics of drugs being tested to treat COVID-19 for hospitalized patients in Tehran.

Variables		Total (*n* = 5318)	Survivor (*n* = 4204)	Deceased (*n* = 1112)	Cramer's V/Eta	*p* value
Plasmapheresis	No	5241 (98.55)	4159 (98.93)	1080 (97.12)	0.06	<0.001
	Yes	76 (1.43)	45 (1.07)	31 (2.79)		
Amantadine	No	5308 (99.81)	4195 (99.79)	1111 (99.91)	0.01	0.396
	Yes	10 (0.19)	9 (0.21)	1 (0.09)		
Acetylsalicylic acid	No	3384 (63.63)	2750 (65.41)	632 (56.83)	0.07	<0.001
	Yes	1927 (36.24)	1451 (34.51)	476 (42.81)		
Atazanavir	No	5232 (98.38)	4140 (98.48)	1090 (98.02)	0.02	0.284
	Yes	86 (1.62)	64 (1.52)	22 (1.98)		
Atorvastatin	No	2996 (56.34)	2430 (57.80)	564 (50.72)	0.06	<0.001
	Yes	2277 (42.82)	1738 (41.34)	539 (48.47)		
Atrovent	No	5091 (95.73)	4028 (95.81)	1061 (95.41)	0.01	0.535
	Yes	226 (4.25)	175 (4.16)	51 (4.59)		
Azithromycin	No	3147 (59.18)	2386 (56.76)	760 (68.35)	0.1	<0.001
	Yes	2124 (39.94)	1780 (42.34)	343 (30.85)		
Bromhexine	No	5040 (94.77)	3970 (94.43)	1068 (96.04)	0.03	0.032
	Yes	278 (5.23)	234 (5.57)	44 (3.96)		
Calcium carbonate	No	5063 (95.20)	4027 (95.79)	1034 (92.99)	0.05	<0.001
	Yes	253 (4.76)	176 (4.19)	77 (6.92)		
Ceftriaxone	No	2761 (51.92)	2124 (50.52)	636 (57.19)	0.05	<0.001
	Yes	2555 (48.04)	2078 (49.43)	476 (42.81)		
Celexan	No	3318 (62.39)	2553 (60.73)	764 (68.71)	0.07	<0.001
	Yes	2000 (37.61)	1651 (39.27)	348 (31.29)		
Clindamycin	No	5100 (95.90)	4049 (96.31)	1049 (94.33)	0.05	0.001
	Yes	178 (3.35)	123 (2.93)	55 (4.95)		
Ciprofloxacin	No	4942 (92.93)	3975 (94.55)	965 (86.78)	0.12	<0.001
	Yes	376 (7.07)	229 (5.45)	147 (13.22)		
Clidinium C	No	5302 (99.70)	4190 (99.67)	1110 (99.82)	0.01	0.407
	Yes	16 (0.30)	14 (0.33)	2 (0.18)		
Combivent	No	4834 (90.90)	3834 (91.20)	999 (89.84)	0.02	0.121
	Yes	442 (8.31)	336 (7.99)	105 (9.44)		
Dexamethasone	No	2892 (54.38)	2338 (55.61)	554 (49.82)	0.05	0.001
	Yes	2382 (44.79)	1832 (43.58)	548 (49.28)		
Dextromethorphan	No	4999 (94.00)	3944 (93.82)	1053 (94.69)	0.02	0.277
	Yes	278 (5.23)	227 (5.40)	51 (4.59)		
Dimenhydrinate	No	5235 (98.44)	4133 (98.31)	1100 (98.92)	0.03	0.06
	Yes	43 (0.81)	39 (0.93)	4 (0.36)		
Diphenhydramin	No	3802 (71.49)	2945 (70.05)	856 (76.98)	0.06	<0.001
	Yes	1471 (27.66)	1224 (29.12)	246 (22.12)		
Fluconazole	No	5234 (98.42)	4158 (98.91)	1074 (96.58)	0.08	<0.001
	Yes	82 (1.54)	45 (1.07)	37 (3.33)		
Heparin	No	2745 (51.62)	2323 (55.26)	421 (37.86)	0.14	<0.001
	Yes	2570 (48.33)	1879 (44.70)	690 (62.05)		
Hydroxychloroquine	No	3061 (57.56)	2411 (57.35)	649 (58.36)	0.01	0.766
	Yes	1086 (20.42)	851 (20.24)	235 (21.13)		
Imipenem	No	5067 (95.28)	4057 (96.50)	1008 (90.65)	0.11	<0.001
	Yes	251 (4.72)	147 (3.50)	104 (9.35)		
Interferon	No	3176 (59.72)	2551 (60.68)	624 (56.12)	0.04	0.005
	Yes	2088 (39.26)	1610 (38.30)	477 (42.90)		
Kaletra	No	3149 (59.21)	2506 (59.61)	642 (57.73)	0.04	0.005
	Yes	954 (17.94)	719 (17.10)	235 (21.13)		
Levofloxacin	No	4851 (91.22)	3875 (92.17)	975 (87.68)	0.07	<0.001
	Yes	427 (8.03)	297 (7.06)	129 (11.60)		
Linezolid	No	5238 (98.50)	4163 (99.02)	1073 (96.49)	0.09	<0.001
	Yes	79 (1.49)	40 (0.95)	39 (3.51)		
Meropenem	No	3936 (74.01)	3328 (79.16)	606 (54.50)	0.23	<0.001
	Yes	1336 (25.12)	838 (19.93)	498 (44.78)		
Magnesium sulfate	No	4960 (93.27)	3929 (93.46)	1029 (92.54)	0.02	0.263
	Yes	357 (6.71)	274 (6.52)	83 (7.46)		
N-acetyl cysteine	No	4600 (86.50)	3687 (87.70)	911 (81.92)	0.07	<0.001
	Yes	715 (13.44)	514 (12.23)	201 (18.08)		
Ondansetron	No	5009 (94.19)	3943 (93.79)	1064 (95.68)	0.04	0.01
	Yes	266 (5.00)	227 (5.40)	39 (3.51)		
Oseltamivir	No	3711 (69.78)	2907 (69.15)	803 (72.21)	0.04	0.019
	Yes	350 (6.58)	293 (6.97)	57 (5.13)		
Piperacillin	No	5312 (99.89)	4200 (99.90)	1110 (99.82)	0.01	0.454
	Yes	6 (0.11)	4 (0.10)	2 (0.18)		
Plasil	No	5288 (99.44)	4181 (99.45)	1105 (99.37)	0	0.744
	Yes	30 (0.56)	23 (0.55)	7 (0.63)		
Plavix	No	4899 (92.12)	3909 (92.98)	988 (88.85)	0.06	<0.001
	Yes	418 (7.86)	295 (7.02)	123 (11.06)		
Prednisolone	No	4886 (91.88)	3879 (92.27)	1005 (90.38)	0.03	0.048
	Yes	426 (8.01)	321 (7.64)	105 (9.44)		
Promethazine	No	5219 (98.14)	4124 (98.10)	1093 (98.29)	0.01	0.67
	Yes	99 (1.86)	80 (1.90)	19 (1.71)		
Pulmi	No	4517 (84.94)	3585 (85.28)	932 (83.81)	0.02	0.229
	Yes	797 (14.99)	616 (14.65)	179 (16.10)		
Ranitidine	No	5055 (95.05)	4006 (95.29)	1047 (94.15)	0.02	0.141
	Yes	261 (4.91)	197 (4.69)	64 (5.76)		
Remdesivir	No	4482 (84.28)	3611 (85.89)	870 (78.24)	0.09	<0.001
	Yes	836 (15.72)	593 (14.11)	242 (21.76)		
Ribavirin	No	4013 (75.46)	3163 (75.24)	849 (76.35)	0.07	<0.001
	Yes	13 (0.24)	4 (0.10)	9 (0.81)		
Salb	No	5189 (97.57)	4113 (97.84)	1074 (96.58)	0.03	0.014
	Yes	128 (2.41)	90 (2.14)	38 (3.42)		
Selenium	No	5159 (97.01)	4078 (97.00)	1079 (97.03)	0	0.959
	Yes	159 (2.99)	126 (3.00)	33 (2.97)		
Seroflo	No	5142 (96.69)	4056 (96.48)	1084 (97.48)	0.02	0.104
	Yes	175 (3.29)	147 (3.50)	28 (2.52)		
Sovodac	No	3993 (75.08)	3141 (74.71)	851 (76.53)	0.01	0.618
	Yes	59 (1.11)	48 (1.14)	11 (0.99)		
Vanco	No	3963 (74.52)	3409 (81.09)	552 (49.64)	0.29	<0.001
	Yes	1350 (25.39)	792 (18.84)	558 (50.18)		
Vitamin B	No	4722 (88.79)	3776 (89.82)	945 (84.98)	0.06	<0.001
	Yes	593 (11.15)	427 (10.16)	165 (14.84)		
Vitamin C	No	3866 (72.70)	3059 (72.76)	806 (72.48)	0	0.824
	Yes	1449 (27.25)	1142 (27.16)	306 (27.52)		
Vitamin D	No	3742 (70.36)	2974 (70.74)	767 (68.97)	0.02	0.245
	Yes	1570 (29.52)	1225 (29.14)	344 (30.94)		
Pantazole	No	1327 (24.95)	1081 (25.71)	246 (22.12)	0.05	0.001
	Yes	2419 (45.49)	1860 (44.24)	558 (50.18)		
Concor (bisoprolol)	No	3212 (60.40)	2561 (60.92)	650 (58.45)	0.1	<0.001
	Yes	448 (8.42)	300 (7.14)	148 (13.31)		
Amlodipine	No	3214 (60.44)	2544 (60.51)	669 (60.16)	0.06	<0.001
	Yes	412 (7.75)	293 (6.97)	119 (10.70)		
Aldactone	No	3321 (62.45)	2613 (62.16)	707 (63.58)	0.03	0.063
	Yes	276 (5.19)	204 (4.85)	72 (6.47)		
Lactulose	No	3121 (58.69)	2462 (58.56)	658 (59.17)	0.03	0.04
	Yes	488 (9.18)	365 (8.68)	123 (11.06)		
Carvedilol	No	3497 (65.76)	2740 (65.18)	756 (67.99)	0	0.803
	Yes	83 (1.56)	66 (1.57)	17 (1.53)		
Fentanyl	No	3406 (64.05)	2778 (66.08)	628 (56.47)	0.36	<0.001
	Yes	177 (3.33)	24 (0.57)	152 (13.67)		
Apotel	No	2552 (47.99)	2014 (47.91)	538 (48.38)	0.02	0.192
	Yes	1109 (20.85)	853 (20.29)	255 (22.93)		
Zinc	No	3115 (58.57)	2430 (57.80)	684 (61.51)	0.01	0.52
	Yes	499 (9.38)	395 (9.40)	103 (9.26)		
Insulin	No	2767 (52.03)	2190 (52.09)	576 (51.80)	0.03	0.061
	Yes	966 (18.16)	737 (17.53)	229 (20.59)		
Lasix	No	2708 (50.92)	2222 (52.85)	485 (43.62)	0.15	<0.001
	Yes	1029 (19.35)	701 (16.67)	328 (29.50)		
Hematinic	No	3499 (65.80)	2735 (65.06)	763 (68.62)	0.03	0.106
	Yes	72 (1.35)	62 (1.47)	10 (0.90)		

The Cramer's V test was used to measure the association between categorical variables and status. The value of Cramer's V indicates how strongly two categorical variables are associated, giving a value between 0 and +1. For numeric variables, the Mann–Whitney test was used to compare median values between survivors and deceased cases. Eta was used to measure the association of numeric variables with status, giving a value between 0 and 1. In both Cramer's V and Eta, values close to 1 indicating a high degree of association. The missing values were ignored in calculation of percentages. The median (Q1, Q3) and frequency (%) were used for describing the numeric and categorical variables, respectively.

## Data Availability

Some restrictions apply to the availability of these data, which were used under license for the current study, and so are not publicly available. Data are however available from the corresponding author on reasonable request.

## Abbreviations

HTN: Hypertension
IHD: Ischemic heart disease
CABG: Coronary artery bypass graft
CHF: Congestive heart failure
COPD: Chronic obstructive pulmonary disease
DM: Diabetes mellitus
CVA: Cerebrovascular accident
CKD: Chronic kidney disease
HLP: Hyperlipidemia
WBC: White blood cell
PLT: Platelets
Hb: Hemoglobin
MCV: Mean corpuscular volume
Cr: Creatinine
AST: Aspartate aminotransferase
ALT: Alanine transaminase
LDH: Lactate dehydrogenase
CRP: C-reactive protein
Na: Sodium
K: Potassium
Ca: Calcium
P: Phosphorous
BIL: Bilirubin
TG: Triglyceride
Chol: Cholesterol
HDL: High-density lipase
LDL: Low-density lipase
FBS: Fasting blood sugar
HbA1c: Hemoglobin A1c
ESR: Erythrocyte sedimentation rate
IL-6: Interlukine-6
CPK: Creatine phosphokinase
CK-MB: Creatine kinase-MB
Pro-BNP: N-Terminal pro b-type natriuretic peptide
PTT: Partial thromboplastin time
PT: Prothrombin time
INR: International normalized ratio
BE: Bass excess
ANCA: Antineutrophil cytoplasmic antibodies
c-ANCA: Cytoplasmic antineutrophil cytoplasmic antibodies
p-ANCA: Prenuclear antineutrophil cytoplasmic antibodies
FDP: Fibrinogen-degradation product
SI: Serum iron
TIBCL: Total iron-binding capacity
TSH: Thyroid stimulating hormone
T4: Thyroxine
T3: Triiodothyronine
IgM: Immunoglobulin M
IgG: Immunoglobulin G.

## Supplementary Materials

Supplementary Materials Table S1: laboratory statistics of COVID-19 patients in Tehran. Figure S1: (A) Drugs being tested to treat COVID-19 for hospitalized patients. (B) Frequency of drug during time (note: only drugs that were used more than 250 times were shown. Labels represented the frequency of drugs for survived and deceased patients). Figure S2: the Kaplan-Meier survival time by demographic variables.
