# Supplementary material for: Epidemiology of COVID-19 in Tehran, Iran: A Cohort Study of Clinical Profile, Risk Factors, and Outcomes
Source: Biomed Res Int. 2022 May 10;2022:2350063. doi: 10.1155/2022/2350063 (PMC9113873; doi:10.1155/2022/2350063)
Supplement: Supplementary Materials — Table S1: laboratory statistics of COVID-19 patients in Tehran. Figure S1: (A) Drugs being tested to treat COVID-19 for hospitalized patients. (B) Frequency of drug during time (note: only drugs that were used more than 250 times were shown. Labels represented the frequency of drugs for survived and deceased patients). Figure S2: the Kaplan-Meier survival time by demographic variables. [file 2350063.f1.docx]

Table S1. Laboratory statistics of COVID‐19 patients in Tehran

| **Variables** |  | **Total (n=5318)** | **Survivor (n=4204)** | **Deceased (n=1112)** | **Cramer's V/Eta** | **P-value** |
| --- | --- | --- | --- | --- | --- | --- |
| WBC1 (× 10^3^/μL) | ----- | 7.3 (5.2, 10.5) | 6.9 (5.0, 9.7) | 9.1 (6.2, 13.2) | 0.17 | <0.001 |
| WBC2 (× 10^3^/μL) | ----- | 7.5 (5.1, 10.9) | 6.9 (4.9, 9.8) | 10.0 (6.6, 14.4) | 0.26 | <0.001 |
| WBC3 (× 10^3^/μL) | ----- | 8.2 (5.8, 11.4) | 7.7 (5.5, 10.4) | 10.2 (7.2, 14.4) | 0.25 | <0.001 |
| WBC4 (× 10^3^/μL) | ----- | 8.4 (6.1, 11.5) | 7.9 (5.8, 10.5) | 10.7 (7.6, 14.8) | 0.28 | <0.001 |
| WBC5 (× 10^3^/μL) | ----- | 8.4 (6.2, 11.3) | 8.0 (6.0, 10.6) | 10.2 (7.6, 13.5) | 0.22 | <0.001 |
| WBC6 (× 10^3^/μL) | ----- | 8.5 (6.4, 11.6) | 8.1 (6.1, 10.7) | 10.6 (7.3, 14.1) | 0.23 | <0.001 |
| Lymphs1 (%) | ----- | 15.6 (10.0, 24.9) | 17.9 (11.0, 25.4) | 10.1 (7.1, 17.1) | 0.22 | <0.001 |
| Lymphs2 (%) | ----- | 14.0 (9.0, 21.6) | 15.0 (10.0, 23.4) | 10.0 (6.0, 14.3) | 0.24 | <0.001 |
| Lymphs3 (%) | ----- | 12.0 (8.0, 20.0) | 13.8 (9.7, 21.7) | 8.9 (5.4, 12.7) | 0.23 | <0.001 |
| Lymphs4 (%) | ----- | 12.0 (8.0, 20.0) | 13.7 (9.6, 21.0) | 8.3 (5.0, 12.3) | 0.22 | <0.001 |
| Lymphs5 (%) | ----- | 12.3 (8.0, 20.0) | 14.5 (10.0, 21.0) | 8.3 (5.0, 13.4) | 0.24 | <0.001 |
| Lymphs6 (%) | ----- | 12.4 (8.0, 20.0) | 14.6 (10.0, 21.6) | 8.0 (5.0, 11.7) | 0.27 | <0.001 |
| NEUT1 (%) | ----- | 79.5 (70.0, 85.0) | 76.9 (68.0, 85.0) | 85.0 (77.4, 90.0) | 0.23 | <0.001 |
| NEUT2 (%) | ----- | 81.0 (72.0, 88.0) | 80.0 (70.0, 86.0) | 86.4 (80.0, 90.5) | 0.25 | <0.001 |
| NEUT3 (%) | ----- | 83.0 (74.6, 90.0) | 80.3 (71.3, 88.0) | 88.0 (81.9, 91.2) | 0.23 | <0.001 |
| NEUT4 (%) | ----- | 83.0 (74.0, 89.0) | 80.0 (72.0, 87.1) | 87.6 (82.0, 91.0) | 0.23 | <0.001 |
| NEUT5 (%) | ----- | 82.0 (73.0, 89.0) | 80.0 (70.1, 87.0) | 87.6 (81.1, 91.9) | 0.26 | <0.001 |
| NEUT6 (%) | ----- | 81.7 (72.3, 88.9) | 80.0 (70.0, 86.0) | 88.5 (81.9, 92.0) | 0.28 | <0.001 |
| PLT1 (× 10^3^/μL) | ----- | 194.0 (150.0, 255.0) | 196.0 (152.0, 254.0) | 186.0 (138.5, 259.0) | 0.04 | <0.001 |
| PLT2 (× 10^3^/μL) | ----- | 193.0 (143.0, 260.0) | 197.0 (147.0, 266.0) | 179.0 (132.0, 244.5) | 0.08 | <0.001 |
| PLT3 (× 10^3^/μL) | ----- | 206.0 (150.0, 280.0) | 214.0 (157.0, 289.0) | 182.0 (128.0, 249.0) | 0.14 | <0.001 |
| PLT4 (× 10^3^/μL) | ----- | 221.0 (157.0, 299.0) | 232.0 (168.0, 312.3) | 187.0 (127.0, 254.0) | 0.18 | <0.001 |
| PLT5 (× 10^3^/μL) | ----- | 226.0 (161.0, 314.0) | 241.0 (174.0, 331.3) | 183.0 (126.0, 259.0) | 0.23 | <0.001 |
| PLT6 (× 10^3^/μL) | ----- | 233.0 (169.0, 323.8) | 248.0 (184.0, 337.0) | 185.0 (123.0, 259.0) | 0.23 | <0.001 |
| HB1 (g/dL) | ----- | 12.4 (10.9, 13.7) | 12.5 (11.1, 13.8) | 11.9 (10.1, 13.3) | 0.12 | <0.001 |
| HB2 (g/dL) | ----- | 11.9 (10.5, 13.3) | 12.0 (10.7, 13.3) | 11.6 (9.8, 13.1) | 0.09 | <0.001 |
| HB3 (g/dL) | ----- | 11.6 (10.2, 12.9) | 11.7 (10.3, 13.0) | 11.2 (9.7, 12.7) | 0.10 | <0.001 |
| HB4 (g/dL) | ----- | 11.6 (10.2, 12.9) | 11.8 (10.4, 13.0) | 11.1 (9.5, 12.7) | 0.11 | <0.001 |
| HB5 (g/dL) | ----- | 11.7 (10.3, 13.0) | 11.8 (10.4, 13.0) | 11.1 (9.7, 12.8) | 0.11 | <0.001 |
| HB6 (g/dL) | ----- | 11.7 (10.2, 13.1) | 11.8 (10.4, 13.2) | 11.1 (9.6, 12.8) | 0.12 | <0.001 |
| MCV1 (μm^3^) | ----- | 84.6 (80.5, 88.3) | 84.3 (80.4, 88.0) | 85.7 (80.7, 89.7) | ----- | <0.001 |
| MCV2 (μm^3^) | ----- | 84.8 (81.2, 88.4) | 84.5 (81.1, 87.9) | 86.1 (81.6, 89.8) | ----- | <0.001 |
| MCV3 (μm^3^) | ----- | 84.8 (81.2, 88.4) | 84.5 (81.1, 87.9) | 85.8 (81.4, 89.6) | ----- | <0.001 |
| MCV4 (μm^3^) | ----- | 84.9 (81.2, 88.4) | 84.6 (81.1, 88.0) | 85.9 (81.6, 89.7) | ----- | <0.001 |
| MCV5 (μm^3^) | ----- | 84.8 (81.2, 88.3) | 84.6 (81.1, 87.9) | 85.9 (81.7, 89.6) | ----- | <0.001 |
| MCV6 (μm^3^) | ----- | 84.6 (81.1, 88.3) | 84.4 (81.1, 87.9) | 85.8 (81.3, 89.6) | ----- | <0.001 |
| BUN1 (mg/dL) | ----- | 19.0 (13.0, 31.0) | 17.0 (12.0, 26.0) | 29.0 (18.3, 48.8) | 0.29 | <0.001 |
| BUN2 (mg/dL) | ----- | 20.0 (14.0, 33.0) | 18.0 (13.0, 26.0) | 32.0 (21.0, 53.0) | 0.34 | <0.001 |
| BUN3 (mg/dL) | ----- | 21.0 (15.0, 34.0) | 20.0 (14.0, 27.0) | 35.0 (23.0, 54.3) | 0.34 | <0.001 |
| BUN4 (mg/dL) | ----- | 22.0 (15.0, 33.0) | 20.0 (14.0, 28.0) | 35.0 (22.8, 52.3) | 0.33 | <0.001 |
| BUN5 (mg/dL) | ----- | 22.0 (16.0, 34.0) | 21.0 (15.0, 29.0) | 35.0 (22.8, 56.0) | 0.35 | <0.001 |
| BUN6 (mg/dL) | ----- | 23.0 (16.0, 35.0) | 21.0 (15.0, 30.0) | 35.0 (22.5, 54.5) | 0.33 | <0.001 |
| CR1 (mg/dL) | ----- | 1.1 (1.0, 1.5) | 1.1 (0.9, 1.4) | 1.4 (1.1, 2.2) | 0.19 | <0.001 |
| CR2 (mg/dL) | ----- | 1.2 (1.0, 1.5) | 1.1 (0.9, 1.4) | 1.5 (1.1, 2.2) | 0.18 | <0.001 |
| CR3 (mg/dL) | ----- | 1.1 (0.9, 1.5) | 1.1 (0.9, 1.4) | 1.4 (1.0, 2.2) | 0.17 | <0.001 |
| CR4 (mg/dL) | ----- | 1.1 (0.9, 1.4) | 1.1 (0.9, 1.3) | 1.4 (1.0, 2.2) | 0.18 | <0.001 |
| CR5 (mg/dL) | ----- | 1.1 (0.9, 1.4) | 1.1 (0.9, 1.3) | 1.3 (1.0, 2.3) | 0.21 | <0.001 |
| CR6 (mg/dL) | ----- | 1.1 (0.9, 1.4) | 1.0 (0.9, 1.3) | 1.3 (1.0, 2.2) | 0.22 | <0.001 |
| NA (mEq/L) | ----- | 138.0 (135.0, 141.0) | 138.0 (135.0, 140.0) | 138.0 (135.0, 141.0) | 0.04 | 0.031 |
| K (mEq/L) | ----- | 4.1 (3.8, 4.4) | 4.1 (3.8, 4.4) | 4.2 (3.9, 4.7) | 0.13 | <0.001 |
| CA (mg/dL) | ----- | 8.6 (8.1, 9.3) | 8.7 (8.2, 9.3) | 8.5 (8.0, 9.1) | 0.09 | <0.001 |
| MG (mEq/L) | ----- | 1.9 (1.7, 2.2) | 1.9 (1.7, 2.1) | 2.0 (1.8, 2.2) | 0.08 | <0.001 |
| P (mg/dL) | ----- | 3.5 (2.9, 4.1) | 3.4 (2.9, 4.0) | 3.8 (3.1, 4.7) | 0.22 | <0.001 |
| AST1 (U/L) | ----- | 36.0 (24.0, 55.0) | 34.0 (23.4, 50.0) | 44.9 (29.0, 72.0) | 0.09 | <0.001 |
| AST2 (U/L) | ----- | 39.8 (27.0, 64.3) | 37.0 (26.0, 57.8) | 53.0 (32.0, 86.0) | 0.14 | <0.001 |
| AST3 (U/L) | ----- | 44.0 (29.0, 77.0) | 40.0 (28.0, 68.0) | 63.0 (35.3, 138.5) | 0.15 | <0.001 |
| AST4 (U/L) | ----- | 42.0 (27.0, 65.0) | 38.0 (26.5, 58.0) | 57.0 (35.5, 123.0) | 0.18 | <0.001 |
| AST5 (U/L) | ----- | 46.0 (30.0, 74.0) | 41.5 (28.0, 66.0) | 60.0 (39.8, 124.5) | 0.21 | <0.001 |
| AST6 (U/L) | ----- | 43.0 (30.0, 71.0) | 42.0 (29.0, 66.0) | 51.0 (32.5, 100.0) | 0.15 | 0.011 |
| ALT1 (U/L) | ----- | 28.0 (18.0, 46.0) | 27.1 (18.0, 45.0) | 30.0 (18.0, 50.4) | 0.07 | 0.021 |
| ALT2 (U/L) | ----- | 29.8 (19.0, 52.0) | 29.0 (19.0, 51.0) | 31.0 (18.0, 55.6) | 0.16 | 0.398 |
| ALT3 (U/L) | ----- | 39.0 (24.0, 77.0) | 37.4 (23.0, 76.0) | 46.0 (27.5, 91.0) | 0.13 | 0.071 |
| ALT4 (U/L) | ----- | 40.0 (24.0, 72.0) | 40.0 (24.0, 72.0) | 40.0 (23.5, 79.5) | 0.12 | 0.590 |
| ALT5 (U/L) | ----- | 45.0 (27.0, 87.5) | 44.0 (27.0, 85.0) | 51.5 (26.0, 115.5) | 0.17 | 0.208 |
| ALT6 (U/L) | ----- | 50.0 (27.0, 93.8) | 51.0 (29.5, 92.0) | 47.0 (23.0, 102.0) | 0.11 | 0.399 |
| ALKP (U/L) | ----- | 185.0 (138.0, 257.0) | 181.0 (136.0, 248.0) | 205.0 (148.0, 287.0) | 0.12 | <0.001 |
| BILLT (mg/dL) | ----- | 0.8 (0.6, 1.1) | 0.8 (0.6, 1.1) | 0.9 (0.6, 1.2) | 0.11 | <0.001 |
| BILLD (mg/dL) | ----- | 0.3 (0.2, 0.4) | 0.3 (0.2, 0.4) | 0.4 (0.2, 0.5) | 0.13 | <0.001 |
| AMYLASE (U/L) | ----- | 53.0 (38.8, 76.8) | 54.0 (40.0, 75.8) | 49.9 (34.0, 80.0) | 0.0 | 0.164 |
| LIPASE (U/L) | ----- | 26.0 (19.0, 38.0) | 26.0 (19.0, 38.0) | 25.0 (17.6, 38.0) | 0.01 | 0.559 |
| TG (mg/dL) | ----- | 120.0 (90.0, 168.0) | 119.0 (90.0, 168.0) | 123.0 (87.8, 173.0) | 0.01 | 0.957 |
| CHOLESTROL (mg/dL) | ----- | 130.0 (106.0, 158.0) | 133.5 (110.0, 161.0) | 119.5 (96.8, 148.0) | 0.14 | <0.001 |
| HDL (mg/dL) | ----- | 31.0 (28.0, 40.0) | 32.0 (28.0, 40.0) | 30.1 (26.0, 38.0) | 0.04 | 0.053 |
| LDL (mg/dL) | ----- | 73.0 (54.0, 95.0) | 75.0 (58.0, 98.0) | 65.0 (48.0, 84.0) | 0.14 | <0.001 |
| FBS (mg/dL) | ----- | 135.0 (104.0, 194.0) | 131.0 (103.0, 188.0) | 146.0 (109.8, 207.3) | 0.06 | 0.001 |
| HBA1C (% of total Hb) | ----- | 7.5 (6.4, 9.9) | 7.5 (6.4, 10.0) | 7.6 (6.4, 9.5) | 0.03 | 0.527 |
| ALBUMIN (g/dL) | ----- | 3.8 (3.4, 4.2) | 3.9 (3.5, 4.3) | 3.5 (3.1, 3.9) | 0.28 | <0.001 |
| LDH1 (U/L) | ----- | 576.0 (439.0, 800.0) | 547.5 (421.8, 745.0) | 711.0 (520.5, 1072.0) | 0.24 | <0.001 |
| LDH2 (U/L) | ----- | 652.0 (499.5, 880.5) | 614.0 (471.0, 818.0) | 836.0 (584.5, 1224.0) | 0.29 | <0.001 |
| LDH3 (U/L) | ----- | 711.5 (510.0, 1001.3) | 644.0 (488.0, 860.0) | 1018.0 (703.0, 1506.0) | 0.36 | <0.001 |
| LDH4 (U/L) | ----- | 677.0 (518.0, 936.0) | 625.0 (487.5, 815.0) | 1034.0 (700.5, 1564.0) | 0.44 | <0.001 |
| LDH5 (U/L) | ----- | 706.0 (541.5, 994.0) | 652.5 (503.3, 881.5) | 1115.0 (684.5, 1478.0) | 0.36 | <0.001 |
| LDH6 (U/L) | ----- | 704.0 (541.0, 1017.0) | 646.0 (508.8, 873.8) | 1036.0 (708.0, 1600.0) | 0.37 | <0.001 |
| CRP1 (mg/L) | ----- | 29.7 (10.5, 69.1) | 26.8 (10.0, 64.0) | 43.4 (15.0, 86.0) | ----- | <0.001 |
| CRP2 (mg/L) | ----- | 59.4 (19.3, 118.7) | 52.0 (16.9, 105.1) | 86.0 (30.0, 151.4) | ----- | <0.001 |
| CRP3 (mg/L) | ----- | 46.7 (15.0, 106.6) | 42.7 (13.1, 96.2) | 71.5 (24.0, 156.3) | ----- | <0.001 |
| CRP4 (mg/L) | ----- | 36.0 (14.0, 76.4) | 31.0 (12.5, 66.3) | 59.4 (18.0, 126.9) | ----- | <0.001 |
| CRP5 (mg/L) | ----- | 31.0 (11.1, 66.5) | 27.0 (10.0, 57.5) | 56.5 (26.0, 125.3) | ----- | <0.001 |
| CRP6 (mg/L) | ----- | 23.4 (9.3, 60.0) | 20.5 (8.9, 45.4) | 47.4 (16.9, 114.3) | ----- | <0.001 |
| ESR (mm/h) | ----- | 34.0 (18.0, 56.0) | 32.0 (18.0, 56.0) | 36.0 (20.0, 59.0) | 0.06 | <0.001 |
| LACTATE | ----- | 20.0 (15.0, 27.0) | 19.1 (15.0, 25.9) | 22.0 (16.0, 33.0) | 0.20 | <0.001 |
| IL6 (pg/mL) | ----- | 25.6 (10.9, 70.2) | 18.5 (8.1, 44.8) | 46.6 (16.1, 146.0) | 0.33 | 0.004 |
| CPK (U/L) | ----- | 117.0 (63.0, 257.0) | 108.0 (61.0, 232.0) | 150.0 (77.5, 356.5) | 0.08 | <0.001 |
| CKMB (U/L) | ----- | 21.0 (14.0, 33.0) | 20.0 (14.0, 30.0) | 25.0 (17.0, 45.0) | 0.12 | <0.001 |
| PROBNP (pg/mL) | ----- | 868.0 (173.8, 3792.8) | 469.0 (132.0, 2313.0) | 3200.0 (894.0, 9987.0) | 0.32 | <0.001 |
| Procalcitonin (pg/mL) | ----- | 0.4 (0.2, 1.3) | 0.3 (0.2, 0.9) | 0.9 (0.3, 2.6) | 0.08 | <0.001 |
| PTT (s) | ----- | 30.0 (25.6, 35.0) | 30.0 (25.3, 35.0) | 32.0 (26.7, 38.0) | 0.09 | <0.001 |
| PT (s) | ----- | 13.0 (11.9, 13.7) | 13.0 (11.7, 13.3) | 13.0 (12.4, 14.6) | 0.14 | <0.001 |
| INR | ----- | 1.1 (1.0, 1.2) | 1.1 (1.0, 1.2) | 1.1 (1.0, 1.3) | 0.16 | <0.001 |
| PH | ----- | 7.4 (7.3, 7.4) | 7.4 (7.3, 7.4) | 7.4 (7.3, 7.4) | 0.08 | <0.001 |
| PCO2 (mm Hg) | ----- | 44.3 (38.7, 50.0) | 44.6 (39.3, 50.1) | 42.7 (36.3, 49.8) | 0.04 | <0.001 |
| HCO3 (mEq/L) | ----- | 25.8 (22.7, 28.6) | 26.2 (23.5, 28.9) | 23.8 (20.2, 27.3) | 0.20 | <0.001 |
| BE (mmol/L) | ----- | 1.6 (-1.6, 4.4) | 2.0 (-0.7, 4.6) | -0.4 (-5.2, 3.0) | 0.21 | <0.001 |
| ANCA (AU/mL) | ----- | 1.5 (0.9, 8.8) | 1.6 (1.0, 12.4) | 1.0 (1.0, 1.0) | 0.27 | 0.480 |
| CANCA (AU/mL) | ----- | 2.4 (1.8, 4.0) | 2.1 (1.4, 3.0) | 3.6 (2.7, 6.3) | 0.19 | 0.015 |
| PANCA (AU/mL) | ----- | 2.9 (1.7, 4.5) | 2.9 (1.7, 4.4) | 2.8 (1.7, 4.8) | 0.09 | 0.883 |
| FDP (mug/ml) | ----- | 6.5 (4.0, 12.0) | 5.9 (4.0, 9.4) | 12.0 (6.2, 18.0) | 0.30 | <0.001 |
| Fe (μg/dL) | ----- | 43.0 (25.0, 80.0) | 44.0 (25.0, 79.8) | 38.5 (24.0, 82.5) | 0.00 | 0.509 |
| Ferritin (ng/mL) | ----- | 361.0 (194.0, 639.9) | 340.3 (182.6, 598.6) | 456.3 (257.0, 762.0) | ----- | <0.001 |
| TIBC (μg/dL) | ----- | 260.0 (193.3, 328.3) | 269.0 (202.0, 330.0) | 236.0 (167.0, 309.5) | 0.10 | 0.002 |
| Total Protein (g/dL) | ----- | 5.8 (5.2, 6.5) | 6.1 (5.4, 6.7) | 5.6 (5.0, 6.2) | 0.18 | 0.007 |
| TSH (μIU/mL) | ----- | 1.0 (0.4, 2.0) | 1.1 (0.5, 2.0) | 1.0 (0.4, 1.9) | 0.01 | 0.282 |
| T4 (μg/dL) | ----- | 8.1 (6.4, 9.6) | 8.4 (6.8, 9.8) | 7.1 (5.3, 8.5) | 0.24 | <0.001 |
| T3(ng/dL) | ----- | 0.9 (0.7, 1.1) | 0.9 (0.7, 1.1) | 0.8 (0.6, 1.0) | 0.17 | <0.001 |
| VitD3 (ng/mL) | ----- | 25.1 (15.6, 39.0) | 24.5 (15.5, 38.4) | 27.6 (17.1, 42.2) | 0.04 | 0.027 |
| IgM (g/L) | ----- | 65.5 (38.5, 112.5) | 98.0 (37.8, 127.3) | 59.0 (36.5, 65.3) | 0.34 | 0.052 |
| IgG (g/L) | ----- | 1060.5 (835.0, 1394.5) | 1073.0 (877.8, 1422.0) | 976.5 (700.5, 1256.0) | 0.16 | 0.228 |
| UREA1 (mg/dL) | ----- | 37.4 (26.9, 56.0) | 34.4 (25.0, 48.0) | 57.3 (37.3, 88.8) | 0.33 | <0.001 |
| UREA2 (mg/dL) | ----- | 37.5 (26.0, 60.1) | 33.8 (24.0, 48.6) | 61.0 (41.0, 100.0) | 0.37 | <0.001 |
| UREA3 (mg/dL) | ----- | 39.8 (27.6, 65.9) | 35.0 (25.5, 52.0) | 68.0 (43.0, 102.8) | 0.38 | <0.001 |
| UREA4 (mg/dL) | ----- | 39.0 (26.0, 63.8) | 34.5 (24.6, 49.9) | 73.0 (42.7, 111.3) | 0.45 | <0.001 |
| UREA5 (mg/dL) | ----- | 39.0 (26.0, 63.0) | 34.1 (24.0, 49.5) | 71.7 (45.5, 114.5) | 0.46 | <0.001 |
| UREA6 (mg/dL) | ----- | 39.0 (26.4, 60.1) | 34.7 (24.6, 49.3) | 70.7 (45.1, 111.5) | 0.49 | <0.001 |

Note: The Cramer’s V test was used to measure the association between categorical variables and status. The value of Cramer’s V indicates how strongly two categorical variables are associated, giving a value between 0 and +1. For numeric variables, the Mann-Whitney test was used to compare median values between survivors and deceased cases. Eta was used to measure the association of numeric variables with status, giving a value between 0 and 1. In both Cramer’s V and Eta, values close to 1 indicating a high degree of association. The missing values were ignored in calculation of percentages. The median (Q1, Q3) and frequency (%) were used for describing the numeric and categorical variables, respectively.


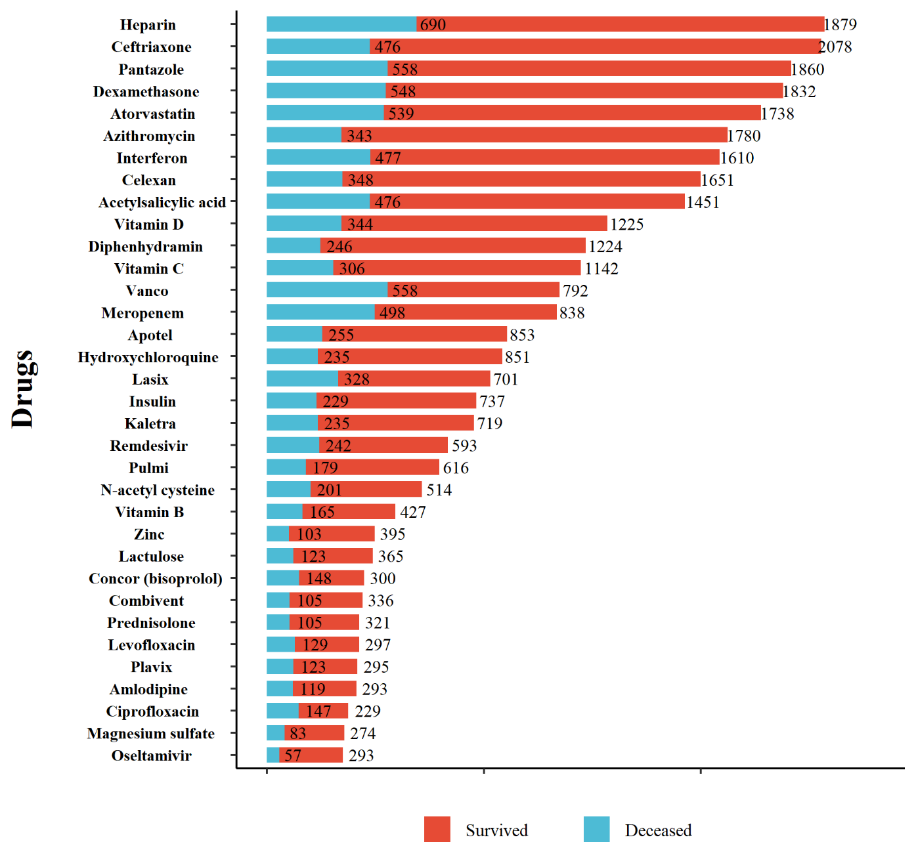


| A |
| --- |


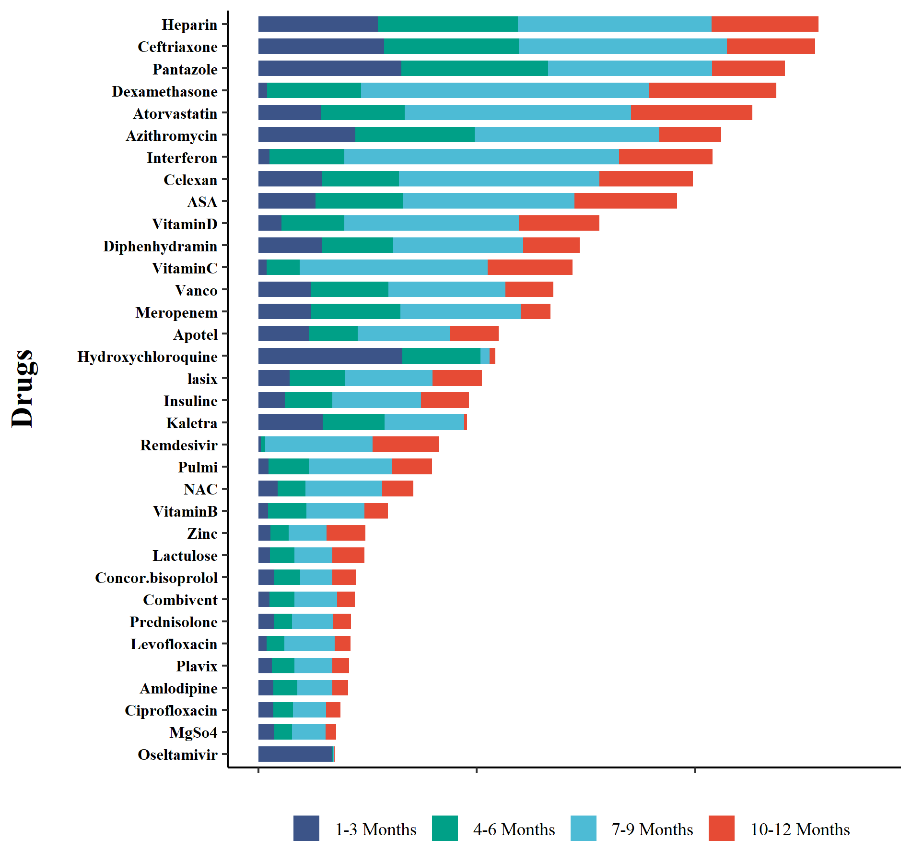


| B |
| --- |

Figure S1. A) Drugs being tested to treat COVID-19 for hospitalized patients, B) Frequency of drug during time (Note: Only drugs that were used more than 250 times were shown. Labels represented the frequency of drugs for survived and deceased patients)


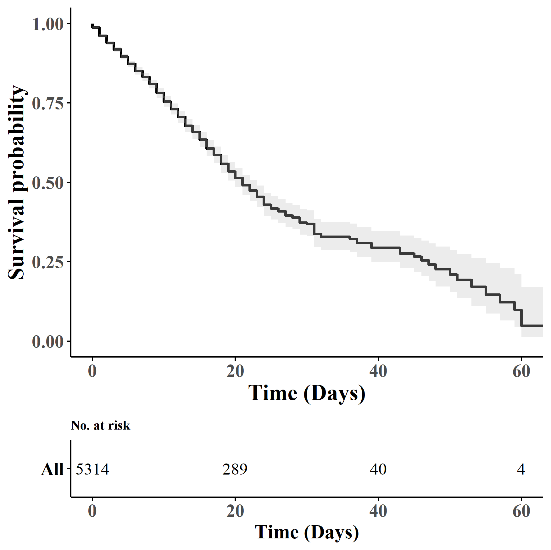

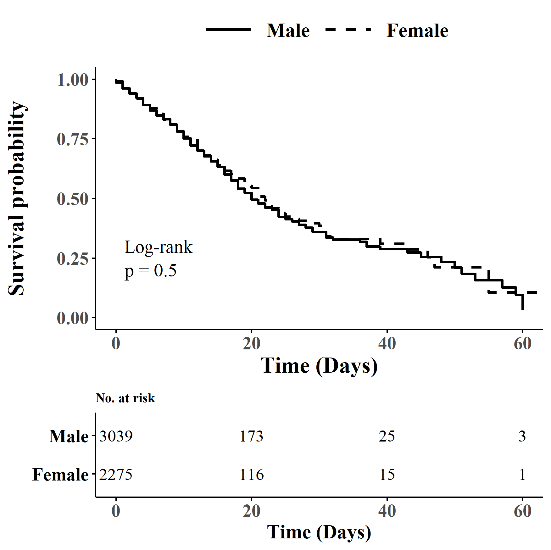

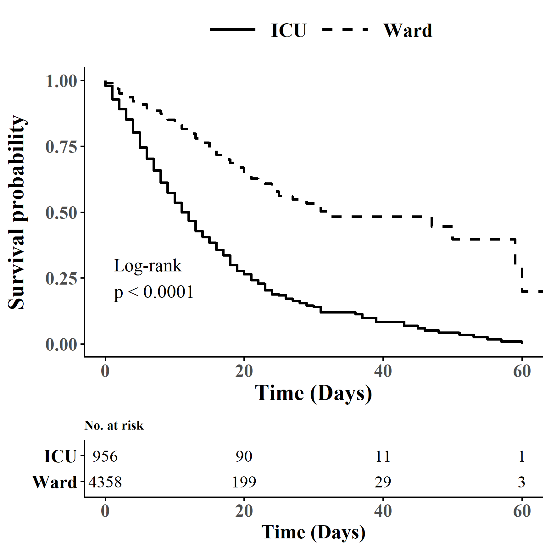


| Overall | Sex | Admission |
| --- | --- | --- |


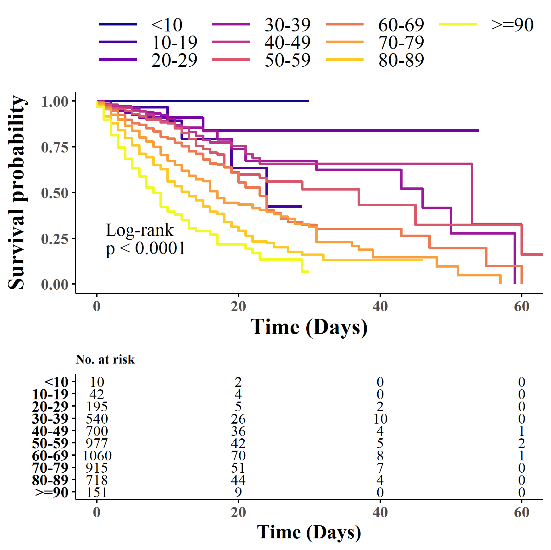

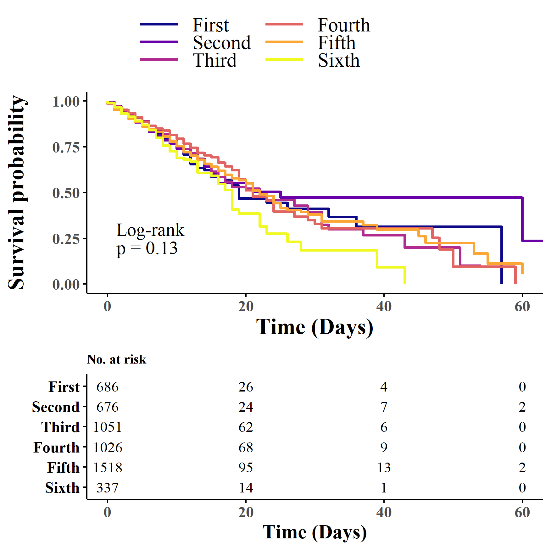

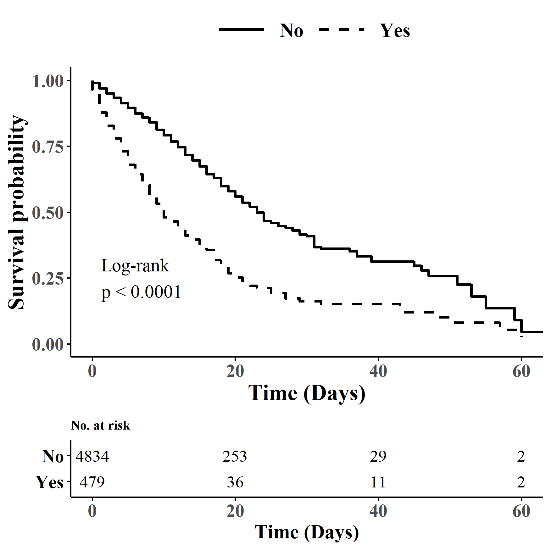


| Age group | Months | Decreased consciousness |
| --- | --- | --- |


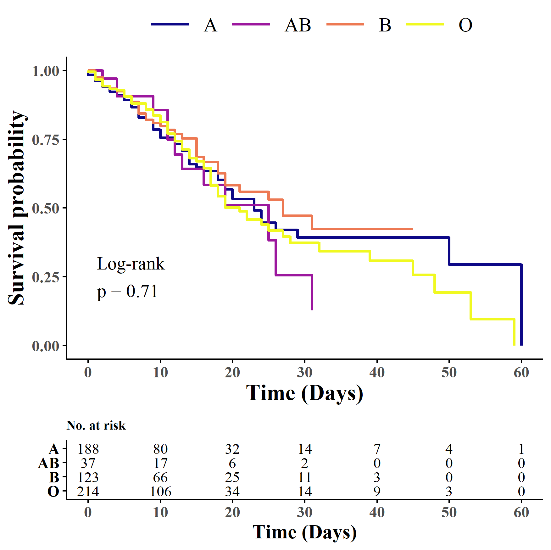

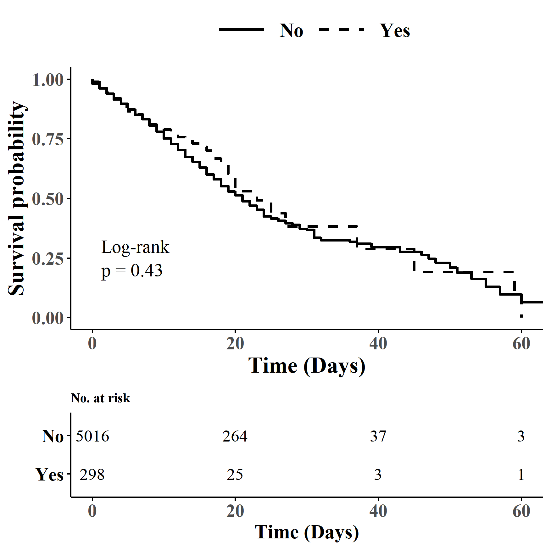

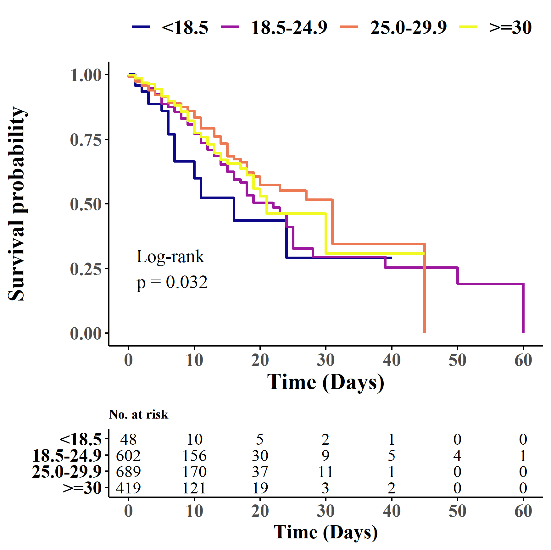


| Blood group | Smoke | BMI |
| --- | --- | --- |

Figure S2. The Kaplan-Meier survival time by demographic variables


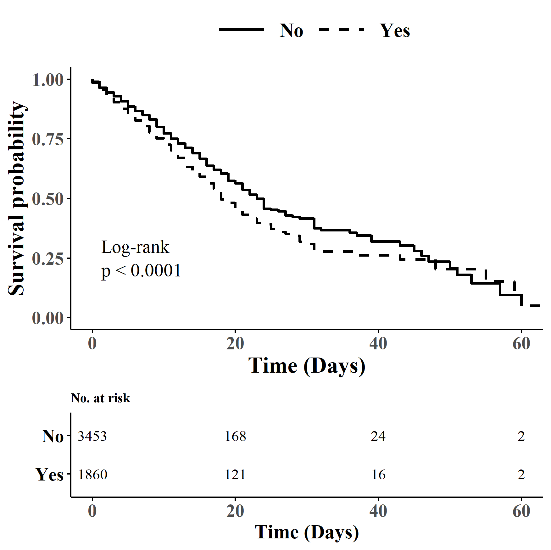

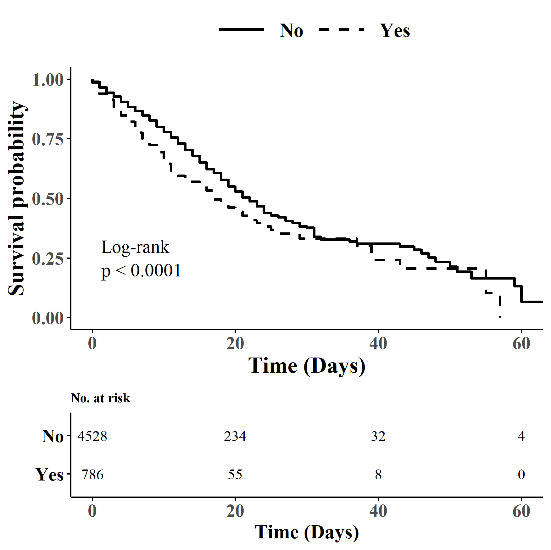

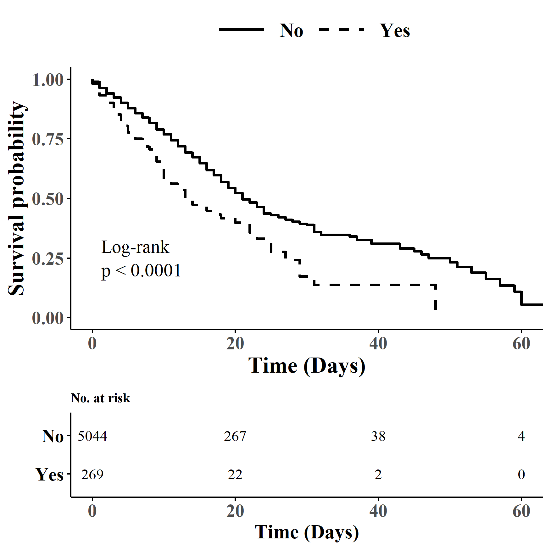


| HTN | IHD | CVA |
| --- | --- | --- |


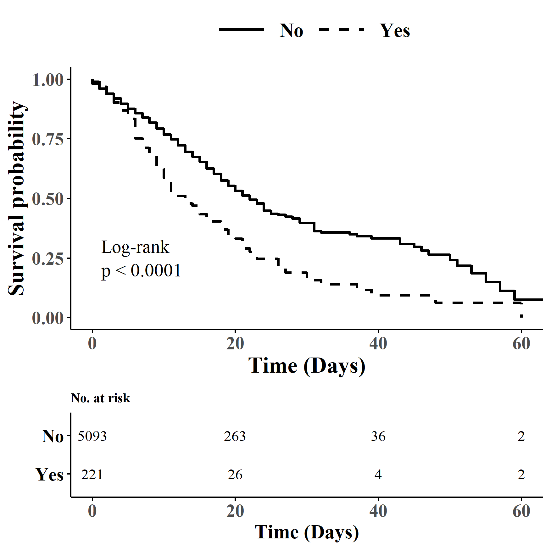

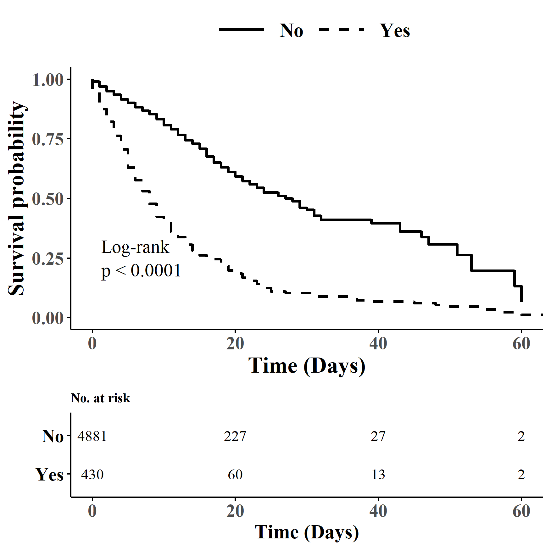

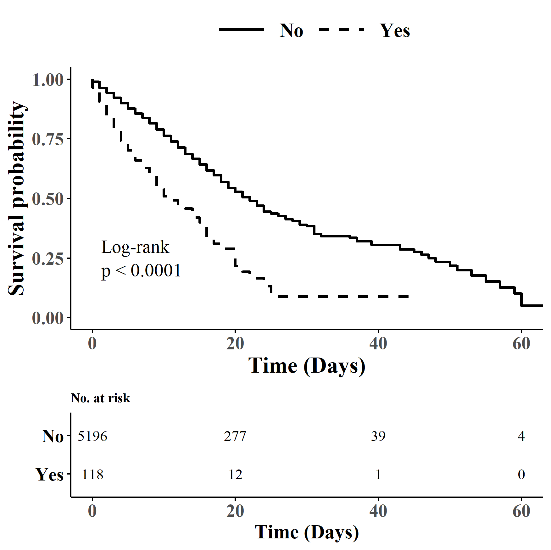


| Dialysis | Intubation | Alzheimer |
| --- | --- | --- |


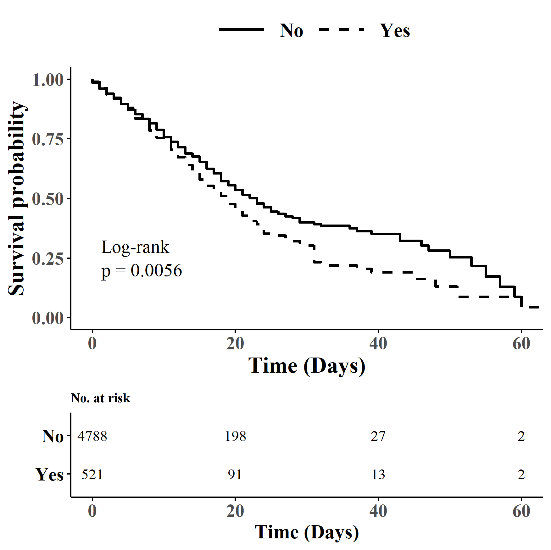

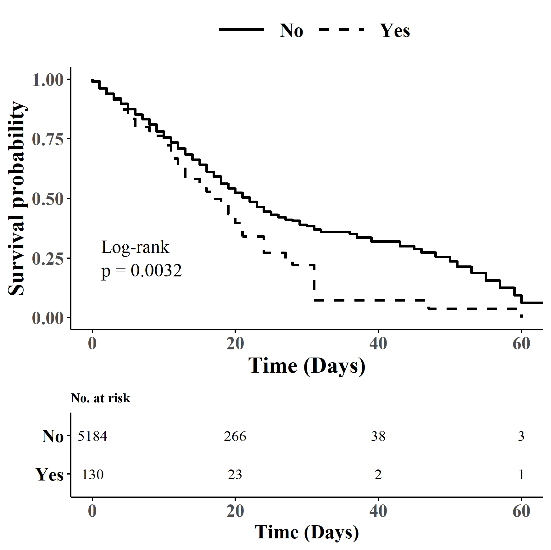

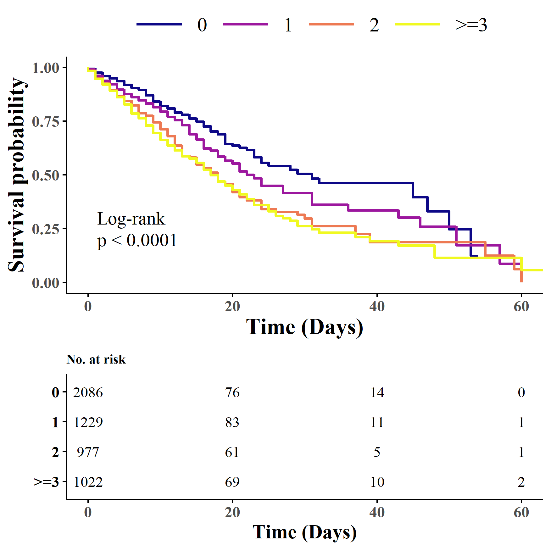


| Blood injection | Injection of platelets or FFP | Number of comorbidities |
| --- | --- | --- |

Figure S2. (Continue) The Kaplan-Meier survival time by demographic variables
